# Cognitive control training with domain-general response inhibition does not change children’s brains or behavior

**DOI:** 10.1038/s41593-024-01672-w

**Published:** 2024-06-04

**Authors:** Keertana Ganesan, Abigail Thompson, Claire R. Smid, Roser Cañigueral, Yongjing Li, Grace Revill, Vanessa Puetz, Boris C. Bernhardt, Nico U. F. Dosenbach, Rogier Kievit, Nikolaus Steinbeis

**Affiliations:** 1 Division of Psychology and Language Sciences, University College London, London, UK; 2 Evidence Based Practice Unit, Anna Freud National Centre for Children and Families, London, UK; 3 Department of Neurology and Neurosurgery, McConnell Brain Imaging Centre, McGill University, Montreal, Quebec Canada; 4 Mallinckrodt Institute of Radiology, Washington University School of Medicine, St. Louis, MO USA; 5 Department of Neurology, Washington University School of Medicine, St. Louis, MO USA; 6 Department of Biomedical Engineering, Washington University in St. Louis, St. Louis, MO USA; 7 Department of Psychological and Brain Sciences, Washington University in St. Louis, St. Louis, MO USA; 8 Department of Pediatrics, Washington University School of Medicine, St. Louis, MO USA; 9 Department of Cognitive Neuroscience, Donders Institute for Brain, Cognition and Behavior, Radboud University Medical Center, Nijmegen, The Netherlands

**Keywords:** Cognitive control, Attention, Psychology

## Abstract

Cognitive control is required to organize thoughts and actions and is critical for the pursuit of long-term goals. Childhood cognitive control relates to other domains of cognitive functioning and predicts later-life success and well-being. In this study, we used a randomized controlled trial to test whether cognitive control can be improved through a pre-registered 8-week intervention in 235 children aged 6–13 years targeting response inhibition and whether this leads to changes in multiple behavioral and neural outcomes compared to a response speed training. We show long-lasting improvements of closely related measures of cognitive control at the 1-year follow-up; however, training had no impact on any behavioral outcomes (decision-making, academic achievement, mental health, fluid reasoning and creativity) or neural outcomes (task-dependent and intrinsic brain function and gray and white matter structure). Bayesian analyses provide strong evidence of absent training effects. We conclude that targeted training of response inhibition does little to change children’s brains or their behavior.

## Abstract (web summary)

Cognitive control is important for later-life success and is often targeted for interventions. Here the authors show that response inhibition training in a large sample of children over 8 weeks did not change their brains or behavior in the short or long term.

## Main

Cognitive control refers to a set of processes critical for guiding thoughts, feelings and actions in a flexible, goal-directed manner^1^. Childhood cognitive control is positively associated with a range of outcomes in other domains, notably social skills^2–6^, academic performance^7,8^ and mental health^9^ and, more crucially, is predictive of these outcomes later in life^7,10,11^. Cognitive control undergoes protracted development from childhood into early adulthood^12–14^. This development is underpinned by the maturation of late-developing fronto-parietal and fronto-striatal neural circuitry^15,16^, supposedly affording extended plasticity^17^. Given its critical role in healthy and productive development, coupled with the prolonged plasticity of its underlying neural circuitry, cognitive control has been a primary target for interventions^18,19^ and particularly so in childhood^20^. Interventions are costly in terms of time, money and opportunity, yet there is continued debate over how successful they actually are.

Cognitive control interventions have primarily focused on improving their hypothesized constituent processes, namely working memory, cognitive flexibility and, to a lesser extent, response inhibition^21,22^. There is broad consensus that these functions can be improved through training, albeit in a relatively narrow and often task-specific manner (that is, near transfer)^23,24^. However, changes in other distally related domains of cognitive functioning and real-world outcomes (that is, far transfer) have been much less consistently observed^22,23,25–32^. Although views differ on whether cognitive training can actually lead to far transfer, the quality of evidence has been consistently questioned^33,34^. Given the likelihood of small effect sizes, criticisms have focused on underpowered samples and poorly specified training mechanisms^33,35,24^. Furthermore, training regimes often lack core features minimally required for far transfer, such as continuously variable, diverse and complex input^18,36,37^, and assessment of training-related outcomes focuses mostly on only short-term effects and a limited number of outcome measures^29^. Finally, the frequent absence of active control groups prohibits drawing any inference on the reasons, let alone mechanisms, for any transfer effects. Here we address whether cognitive control training transfers onto other domains of functioning. We do so in a highly powered sample of children using best practice recommendations for training regimes in terms of diversity, complexity and variability of training input^33,36^ and assessing a wide array of behavioral and neural outcome measures both short and long term.

Unlike most cognitive control interventions, which focus on working memory training^22^, in the present sudy we targeted ‘response inhibition’ as the primary mechanism of action. Inhibition involves a set of highly relevant and widely used processes, including response inhibition or stopping, response selection and contextual monitoring^38^. As such, inhibition may offer a set of cognitive control processes that lend themselves well to training in terms of their domain-general nature as well as the specifically identified training mechanism^39–44^. Using a randomized controlled trial, we assessed the impact of an 8-week cognitive control training with response inhibition as the active ingredient in our experimental group. We compared performance changes on a host of outcome measures with an active control group training response speed, before and after training as well as at a 1-year follow-up. Outcome measures were chosen based on their well-established relationship with cognitive control and response inhibition specifically and included social and intertemporal decision-making^4–6,45^, academic achievement^7,8^, fluid reasoning^46^, mental health (that is, internalizing and externalizing symptoms)^9,47^ as well as creativity^48^. To understand the underlying neurocognitive basis of potential training effects, we also sampled a wide assay of neural indices of brain function, structure and connectivity. In addition to whole-brain analyses, we focused on regions implicated in cognitive control, including the inferior frontal gyrus (IFG)^38,49^ and cingulo-opercular and fronto-parietal networks (CONs and FPNs, respectively)^50^. In addition to assessing the impact of the training regime as a whole, we sought to test two recent hypotheses concerning cognitive control training, namely (1) that far transfer effects emerge only over time^29^ and (2) that near transfer effects mediate far transfer effects^51^. Finally, we made use of the occurrence of a naturally occurring stressor, coronavirus disease 2019 (COVID-19), to test the commonly held view that cognitive control might buffer against the onset of mental health problems^47,52^. Training duration was chosen to be 8 weeks, which was previously shown to be sufficient for far transfer^26,29^.

We developed a highly motivating gamified interface to train response inhibition through variations of the stop-signal task (Experimental Group) or response speed (Control Group). Both groups received identical training in terms of narrative, stimuli and intensity, and the only difference between the groups was how participants were instructed to respond to the stop stimuli (inhibit for the Experimental Group and respond for the Control Group). Training involved a high degree of variation of training contexts and mechanisms and further ensured adaptiveness of the training protocol (Supplementary Figs. 2 and 3) by means of trial-by-trial adaptation (using a staircase procedure) based on performance, such that trials were scaled appropriately to individual abilities for both groups. We refer to closely related domains as ‘near transfer’, which are outcome measures with a highly similar task structure as to what was trained^53^. Everything else we refer to as ‘far transfer’. Power calculations estimated that to obtain even a small Group-by-Session interaction effect of *f* = 0.1 with a power of 0.95 at an alpha Bonferroni corrected for the present number of measures (19; corrected alpha = 0.0025) requires a minimal sample size of 119 participants. The present sample of 235 children is almost twice that and, therefore, amply powered. Leveraging such a large sample also allows us to establish evidence of the absence of the effects of cognitive control training by using Bayesian factor (BF) hypothesis testing^54^. All main hypotheses and analyses for this study were pre-registered: https://osf.io/bn75g/. Correction to control for false discovery rate (FDR) with multiple testing of pre–post training effects was done using the Benjamini–Hochberg procedure^55^.

## Results

### Associations between cognitive control and outcome measures

**Extended Data Fig. 1:**
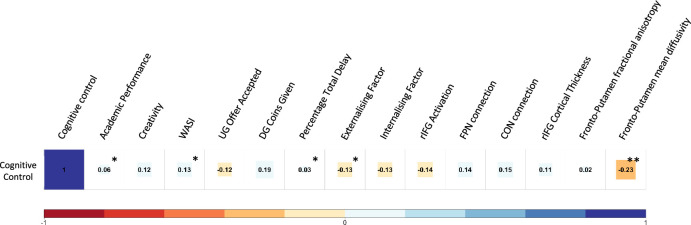
Correlation plots of associations between cognitive control and domains before training. Significant associations were found between cognitive control performance and Academic performance (t(217) = 2.53, *p* = 0.0120, 95%CI = [0.0376, 0.295]), WASI fluid reasoning (t(216) = 2.27, *p* = 0.0240, 95%CI = [0.0203, 0.2800]), delay of gratification (t(226) = 2.44, *p* = 0.015, 95%CI = [0.0310, 0.2843]), externalising symptoms (t(184) = −2.15, *p* = 0.032, 95%CI = [−0.2940, −0.0131]), and mean diffusivity of right fronto-striatal tracts (t(145) = −2.81, *p* = 0.005, 95%CI = [−0.3754, −0.0679]) using one-way.

We first tested how cognitive control performance was associated with each of our outcome measures. To remove task-related variance specific to any assessment of cognitive control, we obtained a single factor of cognitive control derived from multiple cognitive control measures (Methods). We observed significant positive associations between cognitive control performance and several of the outcome measures in the expected direction (Extended Data Fig. 1): delay of gratification (that is, percentage of delayed choices in the intertemporal choice task; *t* (226) = 2.44, *P* = 0.015); academic achievement (*t* (217) = 2.53, *P* = 0.012); fluid reasoning (that is, Wechsler Abbreviated Scale of Intelligence (WASI) scores; *t* (216) = 2.27, *P* = 0.024); externalizing symptoms (*t* (184) = −2.15, *P* = 0.032) as well as mean diffusivity of right fronto-striatal tracts (*t* (145) = −2.81, *P* = 0.005). Cognitive control performance was, thus, correlated with a host of other outcomes, as commonly reported in the literature^7–9,45^.

### Training indices

**Fig. 1:**
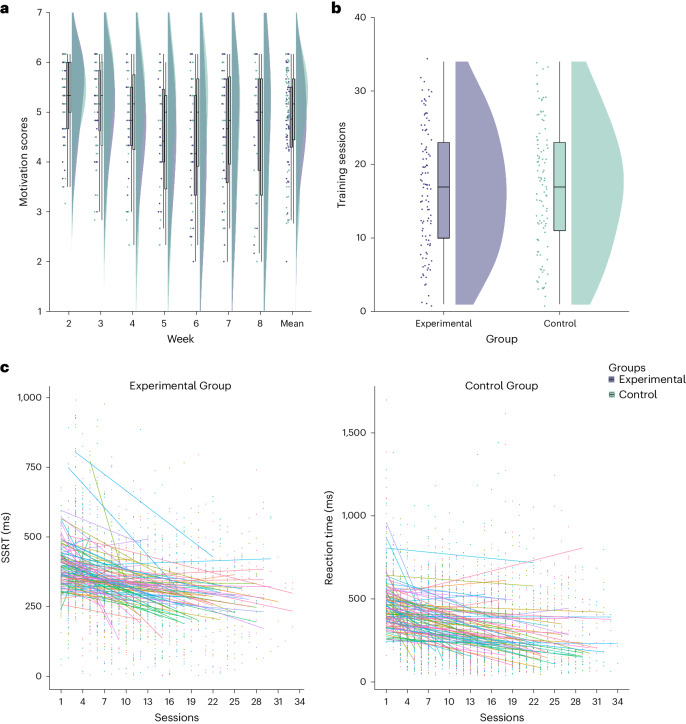
Training metrics. **a**, Motivation (week 2: Experimental Group = 5.30 ± 0.80 (*n* = 38, min = 3.5, max = 6.17, q1–q3 = [4.67,5.67]); Control Group = 5.30 ± 0.72 (*n* = 39, min = 3.17, max = 6.17, q1–q3 = [5,6]) decreased over the weeks *F* (6, 308.75) = 16.42, *P* < 0.001) and was similar (*t* (395.13) = −0.50, *P* = 0.61) between both groups. **b**, Number of sessions (Experimental Group = 16.60 ± 8.35; Control Group = 16.99 ± 8.55) completed was similar (*t* (205.33) = 0.33, *P* > 0.740) between both groups. **c**, Training task performance during training improved in both groups over the course of the training period. Source data

Training took place over an 8-week period. The motivation to train was high to begin with (Experimental Group = 5.30; Control Group = 5.30; out of 1–7) and decreased as training went on (*F* (6, 308.75) = 16.42, *P* < 0.001; Fig. 1a). No group differences were observed in overall motivation between groups (*t* (395.13) = −0.50, *P* = 0.61; BF_10_ = 0.23; Fig. 1a) nor an interaction between Session and Group (*F* (6,308.75) = 1.45, *P* = 0.194). Furthermore, on average, individuals in both groups trained a similar number of sessions (Experimental Group: *n* = 16.60 ± 8.35; Control Group: *n* = 16.99 ± 8.55). No significant difference was observed in the amount trained between both groups (*t* (205.33) = 0.33, *P* > 0.740; BF_10_ = 0.16; Fig. 1b). To assess whether each group improved on the trained cognitive function throughout the intervention, we examined changes over the training sessions in the stop-signal reaction time (SSRT; Experimental Group) and the ‘go-signal’ reaction time (Go RT; Control Group), respectively. For this, we looked at the slope of change in each trained cognitive function using a mixed model with training weeks added as a predictor. There was a main effect of Session where both groups improved on their trained cognitive functions over the training weeks (Experimental Group: *F* (1, 2292.60) = 121.30, *P* < 0.001, η^2^ = 0.05; Control Group: *F* (1, 3197.5) = 185.57, *P* < 0.001, η^2^ = 0.05; Fig. 1c). Thus, groups did not differ in training intensity or motivation and showed moderate improvements during training in the targeted processes.

### Short-term training-related changes

#### Near transfer

**Fig. 2:**
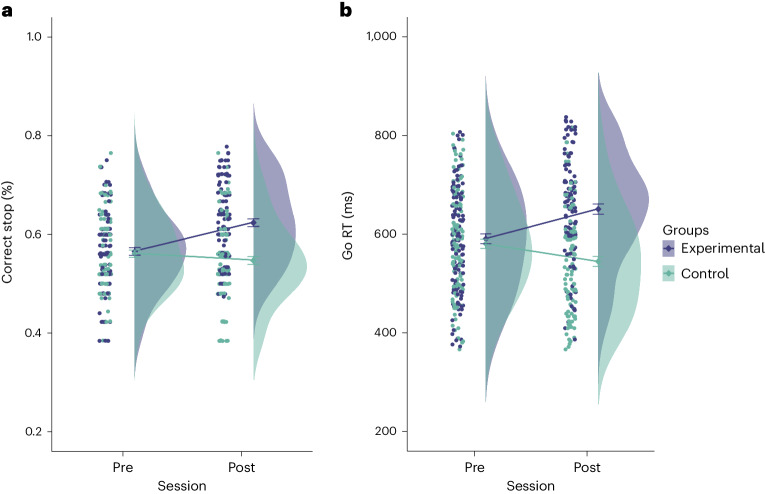
Short-term near transfer. **a**, Percentage correct stop increased significantly in the Experimental Group after training (pre-session: Experimental Group (*n* = 109) = 0.56 ± 0.01, Control Group (*n* = 109) = 0.56 ± 0.01; post-session: Experimental Group (*n* = 107) = 0.62 ± 0.01, Control Group (*n* = 106) = 0.54 ± 0.01). **b**, Go RT increased significantly in the Experimental Group and decreased significantly in the Control Group after training (pre-session: Experimental Group (*n* = 118) = 590.52 ± 9.93, Control Group (*n* = 116) = 580.42 ± 9.42; post-session: Experimental Group (*n* = 109) = 650.63 ± 10.31, Control Group (*n* = 109) = 544.60 ± 10.08). Source data

As a primary measure of near transfer, we looked at the probability of successful stopping and response times to ‘go’ stimuli. The latter is of interest for both indexing training success for the response speed group as well as providing a measure of proactive slowing^56^ for the Experimental Group. A mixed model revealed a significant interaction between Session and Group in the probability of successful stopping in the SSRT (*F* (1,221.00) = 27.31, *P*_*FDRcorr*_ < 0.001, η^2^ = 0.11; Fig. 2a). Follow-up paired *t*-tests comparing pre–post training scores revealed that the probability of successful stopping increased in the Experimental Group (*t* (215) = −5.96, *P* < 0.001). However, no significant change was found in the Control Group (*t* (218) = 1.43, *P* = 0.92). We also observed a significant interaction between Session and Group in Go RT (*F* (1, 227.28) = 31.75, *P*_*FDRcorr*_ < 0.001, η^2^ = 0.12; Fig. 2b). Follow-up paired *t*-tests comparing pre–post training scores revealed that reaction times increased in the Experimental Group (*t* (228) = −5.02, *P* < 0.001) and decreased in the Control Group (*t* (228) = 2.94, *P* = 0.021).

#### Far transfer—behavioral indices

##### Cognitive control

**Fig. 3:**
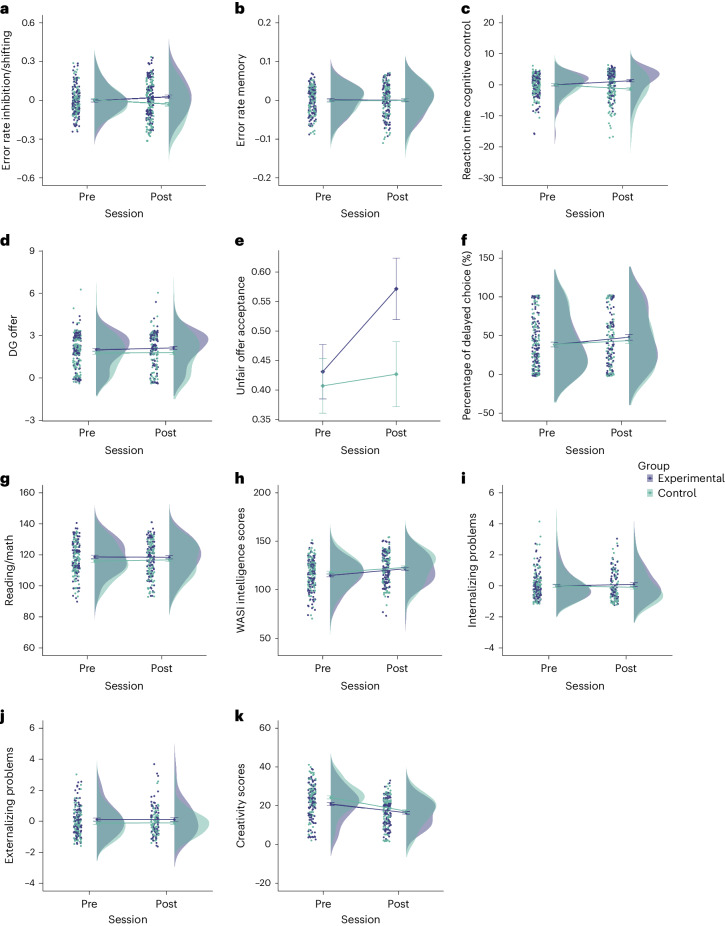
Short-term far transfer on behavioral indices. **a**–**c**, Cognitive control. A significant training effect was found in the inhibition/shifting error factor (pre-session: Experimental Group (*n* = 110) = −0.003 ± 0.01, Control Group (*n* = 107) = 0.004 ± 0.01; post-session: Experimental Group (*n* = 104) = 0.027 ± 0.01, Control Group (*n* = 103) = −0.029 ± 0.01) and the reaction time factor (pre-session: Experimental Group (*n* = 111) = −0.026 ± 0.36, Control Group (*n* = 107) = 0.017 ± 0.30; post-session: Experimental Group (*n* = 104) = 1.31 ± 0.36, Control Group (*n* = 103) = −1.36 ± 0.50). Overall reaction times across all cognitive control tasks increased in the Experimental Group and decreased in the Control Group (error rate memory: pre-session: Experimental Group (*n* = 110) = 0.001 ± 0.003, Control Group (*n* = 107) = −0.001 ± 0.003; post-session: Experimental Group (*n* = 104) = 0.0004 ± 0.004, Control Group (*n* = 103) = −0.0002 ± 0.004). **d**–**f**, Decision-making. DG offer (pre-session: Experimental Group (*n* = 116) = 2.00 ± 0.10, Control Group (*n* = 113) = 1.77 ± 0.11; post-session: Experimental Group (*n* = 91) = 2.13 ± 0.12, Control Group (*n* = 82) = 1.80 ± 0.14); unfair offer acceptance (pre-session: Experimental Group (*n* = 116) = 0.43 ± 0.05, Control Group (*n* = 113) = 0.41 ± 0.05; post-session: Experimental Group (*n* = 91) = 0.57 ± 0.05, Control Group (*n* = 82) = 0.43 ± 0.05); percentage of delayed choice (pre-session: Experimental Group (*n* = 116) = 38.55 ± 3.06, Control Group (*n* = 112) = 38.80 ± 3.21; post-session: Experimental Group (*n* = 89) = 48.0 ± 3.72, Control Group (*n* = 82) = 43.63 ± 3.86). **g**, Academic achievement (pre-session: Experimental Group (*n* = 109) = 118.69 ± 1.02, Control Group (*n* = 110) = 116.01 ± 1.02; post-session: Experimental Group (*n* = 109) = 118.43 ± 1.04, Control Group (*n* = 110) = 116.75 ± 1.04). **h**, Fluid reasoning (pre-session: Experimental Group (*n* = 111) = 114.85 ± 1.45, Control Group (*n* = 107) = 117.66 ± 1.50; post-session: Experimental Group (*n* = 104) = 121.59 ± 1.51, Control Group (*n* = 103) = 123.0 ± 1.56). **i**,**j**, Changes in mental health, separately for internalizing problems (pre-session: Experimental Group (*n* = 96) = −0.008 ± 0.08, Control Group (*n* = 90) = 0.008 ± 0.10; post-session: Experimental Group (*n* = 63) = 0.10 ± 0.12, Control Group (*n* = 68) = −0.091 ± 0.11) and externalizing problems (pre-session: Experimental Group (*n* = 96) = 0.11 ± 0.10, Control Group (*n* = 90) = −0.12 ± 0.09; post-session: Experimental Group (*n* = 63) = 0.11 ± 0.14, Control Group (*n* = 68) = −0.10 ± 0.09). **k**, Creativity (pre-session: Experimental Group (*n* = 111) = 20.94 ± 0.81, Control Group (*n* = 102) = 24.28 ± 0.78; post-session: Experimental Group (*n* = 104) = 16.39 ± 0.67, Control Group (*n* = 98) = 17.20 ± 0.77). Source data

Training cognitive control was operationalized by targeting response inhibition. We assessed the impact of training response inhibition on other subprocesses associated with cognitive control (that is, inhibition as measured by tasks other than the SSRT, shifting and working memory). Given the potentially different impact of training on both speed and accuracy^57^, we performed factor analyses across all cognitive control tasks separately for error rates and reaction times (Methods). This yielded two factors for error rates (one jointly for inhibition and shifting and one for memory) and one single factor for reaction times. For error rates, there was a Session-by-Group interaction found with the inhibition/shifting factor (*F* (1, 215.68) = 10.678, *P*_*FDRcorr*_ = 0.006, η^2^ = 0.05; Fig. 3a). Follow-up paired *t*-tests, however, revealed that neither group changed significantly from pre-training to post-training. For the memory factor, there was no Session-by-Group interaction (*F* (1, 212.72) = 0.090, *P* = 0.764, η^2^ < 0.001, BF_10_ = 0.188; Fig. 3b). For the reaction time factor, there was a significant Session-by-Group interaction (*F* (1,213.71) = 18.60, *P*_*FDRcorr*_ < 0.001, η^2^ = 0.08; Fig. 3c). Pre–post *t*-test comparisons in the Experimental Group revealed an increase from pre-training to post-training (*t* (213) = −2.94, *P* = 0.022) and a decrease for the Control Group (*t* (212) = 3.16 *P* = 0.011).

##### Decision-making

For the role of the proposer in the Dictator Game (DG) for coins shared, there was no significant Session-by-Group interaction (*F* (1, 199.18) = 0.144, *P* = 0.705, η^2^ < 0.001, BF_10_ = 0.201; Fig. 3d). For the role of the responder in the Ultimatum Game (UG) for offers accepted, there was no significant Session-by-Group interaction (*F* (1, 196.49) = 2.36, *P* = 0.126, η^2^ = 0.01, BF_10_ = 0.176; Fig. 3e). In the intertemporal choice task, there was no significant Session-by-Group interaction in the total percentage of delayed choices (*F* (1, 203.60) = 1.01, *P* = 0.317, η^2^ = 0.004, BF_10_ = 0.150; Fig. 3f).

##### Academic performance

There was no significant Session-by-Group interaction for total academic scores (*F* (1, 217.35) = 0.266, *P* = 0.606, η^2^ = 0.001, BF_10_ = 0.159; Fig. 3g).

##### WASI

There was no significant Session-by-Group interaction found for WASI scores (*F* (1, 211.92) = 0.351, *P* = 0.554, η^2^ = 0.001, BF_10_ = 0.169; Fig. 3h).

##### Mental Health

There was no significant Session-by-Group interaction found for either internalizing problems (*F* (1, 125.47) = 4.10, *P* = 0.159, BF_10_ = 0.194; Fig. 3i) or externalizing problems (*F* (1, 123.94) = 0.972, *P* = 0.326, η^2^ = 0.007, BF_10_ = 0.228; Fig. 3j).

##### Creativity

There was no significant Session-by-Group interaction for total creativity scores (a sum score of the five measures from Torrance Tests of Creative Thinking (TTCT); *F* (1, 209.32) = 3.373, *P* = 0.068, η^2^ = 0.02, BF_10_ = 0.448; Fig. 3k).

#### Far transfer—neural indices

##### Functional magnetic resonance imaging

**Fig. 4:**
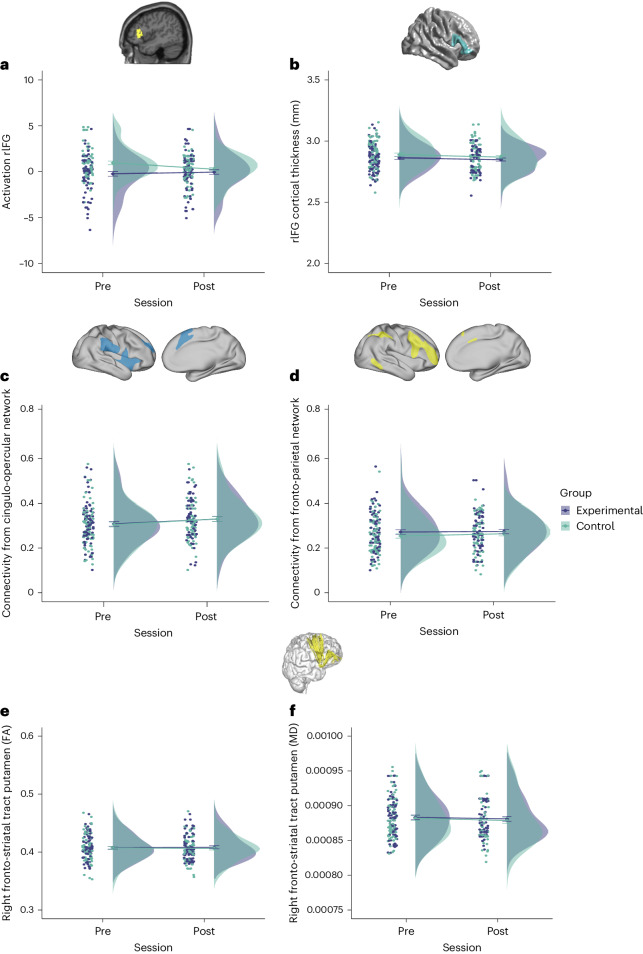
Short term far transfer on neural indices. Changes before and after training in **a**: activation in right IFG (rIFG) (pre-session: Experimental Group (*n* = 72) = −0.24 ± 0.25, Control Group (*n* = 69) = 0.96 ± 0.20; post-session: Experimental Group (*n* = 70) = −0.05 ± 0.25, Control Group (*n* = 66) = 0.27 ± 0.18). **b**, Cortical thickness in rIFG (pre-session: Experimental Group (*n* = 75) = 2.86 ± 0.01, Control Group (*n* = 71) = 2.89 ± 0.01; post-session: Experimental Group (*n* = 70) = 2.85 ± 0.01, Control Group (*n* = 67) = 2.87 ± 0.01). **c**, Functional connectivity in the CON (pre-session: Experimental Group (*n* = 75) = 0.31 ± 0.01, Control Group (*n* = 72) = 0.30 ± 0.01; post-session: Experimental Group (*n* = 70) = 0.33 ± 0.01, Control Group (*n* = 67) = 0.33 ± 0.01). **d**, Functional connectivity in the FPN (pre-session: Experimental Group (*n* = 75) = 0.27 ± 0.01, Control Group (*n* = 72) = 0.25 ± 0.01; post-session: Experimental Group (*n* = 70) = 0.27 ± 0.01, Control Group (*n* = 67) = 0.26 ± 0.01). **e**, Changes in fractional anisotropy of right fronto-striatal structural connectivity (pre-session: Experimental Group (*n* = 75) = 0.41 ± 0.002, Control Group (*n* = 72) = 0.41 ± 0.003; post-session: Experimental Group (*n* = 70) = 0.41 ± 0.002, Control Group (*n* = 67) = 0.41 ± 0.003). **f**, Changes in mean diffusivity of right fronto-striatal structural connectivity (pre-session: Experimental Group (*n* = 75) = 0.00088 ± 0.000003, Control Group (*n* = 72) = 0.00088 ± 0.000003; post-session: Experimental Group (*n* = 70) = 0.00088 ± 0.000003, Control Group (*n* = 67) = 0.00087 ± 0.000004). FA, fractional anisotropy; MD, mean diffusivity. Source data

Although we report brain regions classically implicated in inhibition during successful versus unsuccessful stop trials in our developmental sample (Supplementary Table 7), when looking at the whole brain, no significant interaction was observed between Session and Group for any voxel after correction for multiple comparisons. We also focussed our analysis on the right IFG, a core hub of cognitive control and response inhibition in particular^49^. For the region of interest (ROI) analysis, parameter estimates for each participant were extracted from the right IFG. A mixed model revealed a significant effect of Group (*F* (1, 271) = 11.43, *P* < 0.001, η^2^ = 0.04; higher activation overall for the Control Group compared to the Experimental Group) and no interaction between Session and Group (*F* (1, 271) = 3.87, *P* = 0.050, η^2^ = 0.01, BF_10_ = 1.105; Fig. 4a). Follow-up *t*-test showed no significant change in either group before or after training.

#### Cortical thickness

To assess potential training-related changes in cortical gray matter structure, we looked at the whole brain. There was no significant interaction between Session and Group for any voxel. We also obtained parameter estimates of cortical thickness for each participant extracted from the right IFG. A mixed model revealed no interaction between Session and Group (*F* (1, 139.85) = 0.016, *P* = 0.901, η^2^ < 0.001, BF_10_ = 0.200; Fig. 4b).

##### Resting-state connectivity

We looked at changes in connectivity profiles in circuits known to be implicated in cognitive control and response inhibition^50^, such as CON and FPN. Connectivity in the CON and FPN was extracted for each participant. Mixed models revealed no interaction between Session and Group in either of the two networks (CON: *F* (1, 141.34) = 0.053, *P* = 0.819, η^2^ < 0.001, BF_10_ = 0.180; Fig. 4c; FPN: *F* (1, 143.14) = 0.162, *P* = 0.688, η^2^ = 0.001, BF_10_ = 0.187; Fig. 4d).

##### Diffusion tensor imaging

Fractional anisotropy and mean diffusivity, two measures of white matter microstructure, were extracted from connections between the frontal lobes and striatal areas of the right hemisphere, given their known role in cognitive control and response inhibition^58^. Mixed models revealed no significant interactions between Session and Group in either fractional anisotropy (*F* (1, 141.63) = 0.134, *p* = 0.715, η^2^ < 0.001, BF_10_ = 0.188; Fig. 4e) or mean diffusivity (*F* (1, 144.24) = 0.019, *P* = 0.891, η^2^ < 0.001, BF_10_ = 0.211; Fig. 4f) in the right frontal-striatal putamen.

### Long-term training-related changes

#### Near transfer

**Fig. 5:**
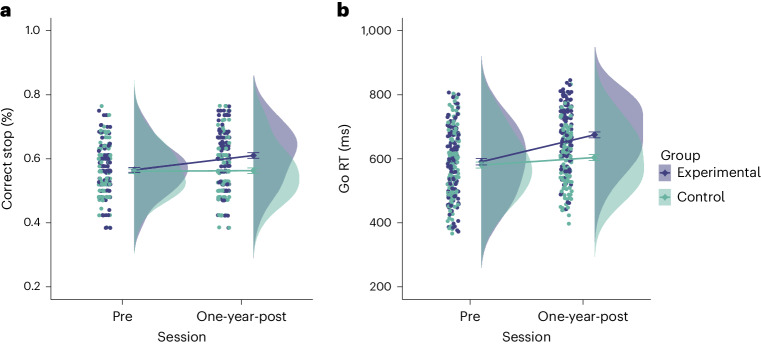
Long-term near transfer. **a**, Percentage correct stop remained increased significantly in the Experimental Group 1 year after training (pre-session: Experimental Group (*n* = 109) = 0.56 ± 0.008, Control Group (*n* = 109) = 0.56 ± 0.007; post-session: Experimental Group (*n* = 107) = 0.61 ± 0.008, Control Group (*n* = 106) = 0.56 ± 0.008). **b**, Go RT remained increased significantly in the Experimental Group 1 year after training (pre-session: Experimental Group (*n* = 118) = 590.52 ± 9.93, Control Group (*n* = 116) = 580.42 ± 9.42; post-session: Experimental Group (*n* = 109) = 674.67 ± 9.18, Control Group (*n* = 109) = 603.75 ± 9.28). Source data

We also tested if any training-related changes might persist or, indeed, emerge over time, as was asserted previously^29^, by comparing performance on outcome measures between training groups 1 year after training. For the probability of successful stopping in the SSRT, there was a significant interaction between Session and Group (*F* (1,227.16) = 8.68, *P*_*FDRcorr*_ = 0.018, η^2^ = 0.04; Fig. 5a). Follow-up paired *t*-tests revealed that the probability of successful stopping remained increased in the Experimental Group (*t* (217) = −4.38, *P* = 0.001) after 1 year; however, no significant change was found in the Control Group (*t* (218) = −0.202, *P* = 1.000). For reaction time to the ‘go’ signal, there was a significant interaction between Session and Group (*F* (1, 235.94) = 13.32, *P*_*FDRcorr*_ < 0.003, η^2^ = 0.05; Fig. 5b). Follow-up paired *t*-tests revealed that reaction times remained elevated in the Experimental Group (*t* (231) = −6.992, *P* < 0.001); however, no significant change was found in the Control Group (*t* (230) = −1.844, *P* = 0.399).

#### Far transfer

##### Cognitive control

**Fig. 6:**
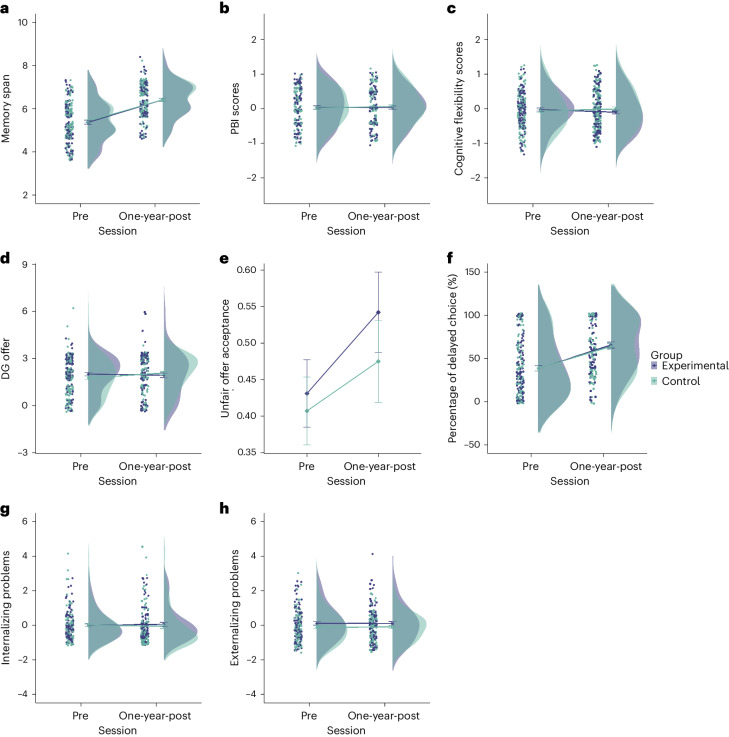
Long-term far transfer. **a**–**c**, Changes in three executive function (EF) tasks 1 year after training: Corsi memory span (pre-session: Experimental Group (*n* = 117) = 5.36 ± 0.085, Control Group (*n* = 116) = 5.42 ± 0.080; post-session: Experimental Group (*n* = 110) = 6.41 ± 0.074, Control Group (*n* = 107) = 6.41 ± 0.071); PBI scores (pre-session: Experimental Group (*n* = 118) = 0.037 ± 0.050, Control Group (*n* = 116) = 0.020 ± 0.044; post-session: Experimental Group (*n* = 110) = 0.030 ± 0.047, Control Group (*n* = 108) = 0.066 ± 0.049); and cognitive flexibility cores (pre-session: Experimental Group (*n* = 114) = −0.024 ± 0.048, Control Group (*n* = 112) = −0.063 ± 0.053; post-session: Experimental Group (*n* = 109) = −0.103 ± 0.053, Control Group (*n* = 108) = −0.010 ± 0.055). **d**–**f**, Changes in three decision-making task variables: DG offer (pre-session: Experimental Group (*n* = 116) = 2.0 ± 0.10, Control Group (*n* = 113) = 1.77 ± 0.11; post-session: Experimental Group (*n* = 83) = 1.94 ± 0.15, Control Group (**n** = 80) = 2.07 ± 0.012); unfair offer acceptance (pre-session: Experimental Group (*n* = 116) = 0.43 ± 0.046, Control Group (*n* = 113) = 0.41 ± 0.046; post-session: Experimental Group (*n* = 83) = 0.54 ± 0.0555, Control Group (*n* = 80) = 0.48 ± 0.056); and percentage of delayed choice (pre-session: Experimental Group (n = 116) = 38.55 ± 3.06, Control Group (*n* = 112) = 38.80 ± 3.21; post-session: Experimental Group (*n* = 82) = 65.58 ± 3.33, Control Group (*n* = 78) = 64.03 ± 3.60). **g**,**h**, Changes in mental health, separated by internalizing problems (pre-session: Experimental Group (*n* = 96) = −0.008 ± 0.08, Control Group (*n* = 90) = 0.008 ± 0.10; post-session: Experimental Group (*n* = 81) = 0.061 ± 0.11, Control Group (*n* = 81) = −0.061 ± 0.13) and externalizing problems (pre-session: Experimental Group (*n* = 96) = 0.11 ± 0.10, Control Group (*n* = 90) = −0.12 ± 0.09; post-session: Experimental Group (*n* = 81) = 0.10 ± 0.12, Control Group (*n* = 81) = −0.10 ± 0.09). Source data

No significant changes remained in executive function tasks 1 year after training. These analyses were performed on a subset of tasks that were carried out at the follow-up due to COVID-19 restrictions. No Session-by-Group interaction was found in memory span in the Corsi task (*F* (1, 228.05) = 0.147, *P* = 0.702, η^2^ < 0.001, BF_10_ = 0.152; Fig. 6a); in proactive control, as measured by the AX-Continuous Performance Task (CPT) (*F* (1, 445) = 0.340, *P* = 0.560, η^2^ < 0.001, BF_10_ = 0.165; Fig. 6b); or in cognitive flexibility (*F* (1, 227.71) = 0.178, *P* = 0.183, η^2^ = 0.008, BF_10_ = 0.294; Fig. 6c).

##### Decision-making

There was no significant Session-by-Group interaction for any of the decision-making measures (sharing in the DG: (*F* (1, 204.53) = 2.74, *P* = 0.099, η^2^ = 0.01, BF_10_ = 0.450; Fig. 6d)); for proportion of accepted offers in the UG (*F* (1, 198.66) = 0.385, *P* = 0.536, η^2^ = 0.002, BF_10_ = 0.174; Fig. 6e); or for percentage delayed choice in the intertemporal choice task (*F* (1, 202.89) = 0.116, *P* = 0.733, η^2^ < 0.001, BF_10_ = 0.166; Fig. 6f).

##### Mental health

No significant Session-by-Group interaction was found for internalizing problems (*F* (1, 154.70) = 2.23, *P* = 0.138, η^2^ = 0.01, BF_10_ = 0.207; Fig. 6g) or externalizing problems (*F* (1, 147.47) = 0.573, *P* = 0.450, η^2^ = 0.004, BF_10_ = 0.162; Fig. 6h).

### Mediation of far transfer by near transfer

A common argument in defense of the large heterogeneity within far transfer effects from training studies is that this depends crucially on whether near transfer is found^51^. We examined if changes in near transfer were in any way predictive of changes in far transfer. Our measure of near transfer was the probability of successful stopping. We found that near transfer was not predictive of performance change on any far transfer measure.

### Training effect on mental health after COVID-19 lockdown

**Extended Data Fig. 2:**
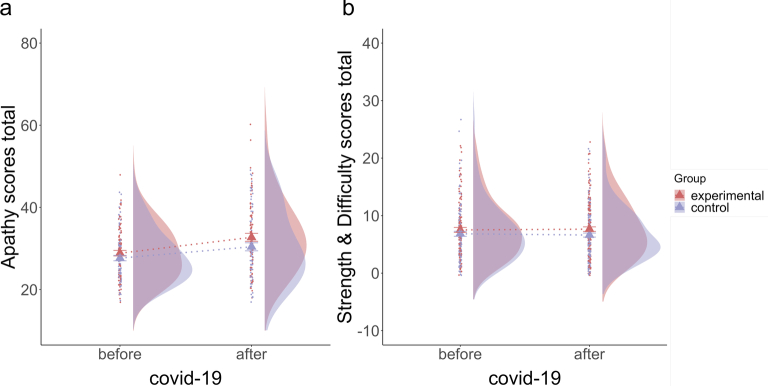
Training effect before and after COVID-19 lockdown. **a**, Changes in Apathy before and after COVID-19 lockdown (pre-COVID: Experimental Group (n = 95) = 28.85 ± 0.67, Control Group (n = 88) = 27.66 ± 0.65; post-COVID: Experimental Group (n = 82) = 32.77 ± 0.94, Control Group (n = 83) = 30.57 ± 0.93). Apathy in both groups increased significantly after lockdown. **b**, Changes in Strength & Difficulties scores (pre-COVID: Experimental Group (n = 95) = 7.53 ± 0.46, Control Group (n = 89) = 6.86 ± 0.51; post-COVID: Experimental Group (n = 82) = 8.15 ± 0.58, Control Group (n = 83) = 6.83 ± 0.50).

Much research has been dedicated to establishing that cognitive control might serve as a buffer to the onset of mental health problems^47,52^. Although our present sample was not at risk, data collection took place during COVID-19, which presented considerable challenges to mental health due to school closures and lockdowns^59^. We examined whether training cognitive control would buffer against any negative impact of COVID-19 measures on mental health. We studied apathy and mental health using the Apathy Evaluation Scale, clinical version (AES-C), and the Strengths & Difficulties Questionnaire (SDQ) for ages 4–17 years before and after the COVID-19 lockdown. We found that both groups were similar in terms of positive cases of COVID-19 as well as perceived stress (Supplementary Tables 5 and 6). Crucially, although we found a significant increase in apathy after the COVID-19 lockdown (*F* (1,178.29) = 29.82, *P* < 0.001; Extended Data Fig. 2a), this was not buffered by response inhibition training (*F* (1, 178.78) = 0.014, *P* = 0.905, η^2^ < 0.001, BF_10_ = 0.188; Extended Data Fig. 2a). There was no buffering effect of training on the strength and difficulties scores after the COVID-19 lockdown (*F* (1, 154.32) = 3.05, *P* = 0.083, η^2^ = 0.008, BF_10_ = 0.141; Extended Data Fig. 2b).

### Controlling for socioeconomic status

To test for the robustness and generalizability of our effects, we re-ran all analyses of short-term and long-term near and far transfer effects while also controlling for socioeconomic status (SES). Controlling for SES did not change any of the outcomes.

## Discussion

The critical role of cognitive control in healthy and productive development and positive later-life outcomes has attracted tremendous interest from researchers and policymakers seeking to understand how cognitive control development can be supported. However, consensus on whether this is possible has been difficult to reach. In this study, we addressed whether cognitive control can be improved by means of a targeted response inhibition training and whether such training has a lasting, wider impact on cognitive and neural functioning. We developed an 8-week intervention, which was administered to a highly powered sample of 235 6–13-year-old children in a pre-registered randomized controlled trial including an active control group training response speed. We found that our training led to specific improvements in the trained functions (that is, response inhibition and response speed), which lasted up to 1 year after training. We further found that response inhibition training led to more cautious responding on a battery of cognitive control tasks. Crucially, however, we did not find any evidence to support the idea that training response inhibition leads to changes in other domains, such as decision-making, academic achievement, fluid reasoning, mental health or creativity. Furthermore, there was no evidence that our training led to any marked changes in brain function, structure or connectivity. There was also no indication of training effects emerging over time, nor did the presence of near transfer effects mediate the likelihood of far transfer. Finally, training response inhibition did not act as a buffer to mental health problems as a result of major social stressors, such as COVID-19. Bayesian tests provide substantial evidence in support of the evidence of absent training effects (Tables 1 and 2). In sum, response inhibition training appears to do little to alter children’s brains or their behavior in long-lasting ways.

**Table 1:** Short-term training effect for each measure with BF values

Domain	df	*F*	*P*	Test	*P* (corrected)	η^2^ (partial)	BF_10_	Interpretation
Correct stop (%)	220.9983	27.31287	4.00 × 10^-7^	SIG	3.80 × 10^-6^	0.11	–	–
Go RT	227.2779	31.7502	5.16 × 10^-8^	SIG	9.81 × 10^-7^	0.12	–	–
Error rate inhibition/shifting	215.6812	10.67843	0.001261	SIG	0.005991	0.05	–	–
Error rate memory	212.7198	0.090184	0.764237	NSIG	0.900727	4.24 × 10^-4^	0.188	Substantial evidence for H0
Reaction time cognitive control	213.7102	18.60236	2.46 × 10^-5^	SIG	0.000156	0.08	–	–
DG offer	199.1767	0.143779	0.704957	NSIG	0.900727	7.21 × 10^-4^	0.201	Substantial evidence for H0
Unfair offer acceptance	196.4909	2.363369	0.125822	NSIG	0.298828	0.01	0.176	Substantial evidence for H0
Percentage of delayed choice	203.6008	1.007366	0.316726	NSIG	0.619391	4.92 × 10^-3^	0.15	Substantial evidence for H0
Reading/math	217.3507	0.266263	0.606374	NSIG	0.900727	1.22 × 10^-3^	0.159	Substantial evidence for H0
WASI intelligence scores	211.9218	0.351003	0.554177	NSIG	0.900727	1.65 × 10^-3^	0.169	Substantial evidence for H0
Internalizing problems	125.4664	4.104408	0.044892	NSIG	0.159122	0.03	0.194	Substantial evidence for H0
Externalizing problems	123.9372	0.972433	0.325995	NSIG	0.619391	7.79 × 10^-3^	0.228	Substantial evidence for H0
Creativity	209.3242	3.373341	0.067677	NSIG	0.183696	0.02	0.448	Anecdotal evidence for H0
Activation right IFG	271	3.867541	0.050249	NSIG	0.159122	0.01	1.105	Anecdotal evidence for H1
Cortical thickness right IFG	139.8476	0.015617	0.900727	NSIG	0.900727	1.12 × 10^-4^	0.2	Substantial evidence for H0
CON connectivity	141.3451	0.05276	0.818661	NSIG	0.900727	3.73 × 10^-4^	0.18	Substantial evidence for H0
FPN connectivity	143.1404	0.161787	0.688117	NSIG	0.900727	1.13 × 10^-3^	0.187	Substantial evidence for H0
Fronto-putamen fractional anisotropy	141.63	0.133993	0.714873	NSIG	0.900727	9.45 × 10^-4^	0.188	Substantial evidence for H0
Fronto-putamen mean diffusivity	144.2376	0.018666	0.89152	NSIG	0.900727	1.29 × 10^-4^	0.211	Substantial evidence for H0

Mixed models were used to examine short-term training effect. Significant interaction effects between Session and Group were interpreted as presence of training-related changes. Effect size was calculated for the interaction effect of Group and Session. The Benjamini–Hochberg procedure was then applied to mixed-model analysis testing for training-related changes. For the results that are significant, we report their adjusted *P* value after correction to control FDR with multiple testing. For evidence of null effects, we report the BF in favor of the null model (the model without a Group-by-Session interaction) over the training model (the model with a Group-by-Session interaction) for each measure of interest. df, degrees of freedom; sig, significant; nsig, non-significant; H0, the degree of change in the outcome measure between the two groups following training is the same; H1, the degree of change in the outcome measure between the two groups following training is different.

**Table 2:** Long-term training effect for each measure with BF values

Domain	df	*F*	*P*	Test	*P* (corrected)	η^2^ (partial)	BF_10_	Interpretation
Correct stop (%)	227.1605	8.683001	0.003547	SIG	0.017733	0.04	–	–
Go RT	235.9409	13.32172	0.000323	SIG	0.00323	0.05	–	–
Memory span	228.0468	0.146533	0.702228	NSIG	0.733347	6.42 × 10^-4^	0.152	Substantial evidence for H0
PBI scores	445	0.339658	0.560322	NSIG	0.700402	7.63 × 10^-4^	0.165	Substantial evidence for H0
Cognitive flexibility scores	227.7137	1.782017	0.183235	NSIG	0.36647	7.76 × 10^-3^	0.294	Substantial evidence for H0
DG offer	204.5271	2.737907	0.099527	NSIG	0.331756	0.01	0.45	Anecdotal evidence for H0
Unfair offer acceptance	198.6605	0.384665	0.535828	NSIG	0.700402	1.93 × 10^-3^	0.174	Substantial evidence for H0
Percentage of delayed choice	202.8912	0.11638	0.733347	NSIG	0.733347	5.73 × 10^-4^	0.166	Substantial evidence for H0
Internalizing problems	154.7039	2.226707	0.137679	NSIG	0.344198	0.01	0.207	Substantial evidence for H0
Externalizing problems	147.4677	0.57311	0.450233	NSIG	0.700402	3.87 × 10^-3^	0.162	Substantial evidence for H0

Mixed models were used to examine long-term training effect. Significant interaction effects between Session and Group were interpreted as presence of training-related changes. Effect size was calculated for the interaction effect of Group and Session. The Benjamini–Hochberg procedure was then applied to mixed-model analysis testing for training-related changes. For the results that are significant, we report their adjusted *P* value after correction to control FDR with multiple testing. For evidence of null effects, we report the BF in favor of the null model (the model without a Group-by-Session interaction) over the training model (the model with a Group-by-Session interaction) for each measure of interest. H0, the degree of change in the outcome measure between the two groups following training is the same; H1, the degree of change in the outcome measure between the two groups following training is different.

Research on the effectiveness of cognitive control interventions has been riddled with contradictory findings^32,60,61^. However, consensus exists that this is best arbitrated by high-quality evidence^33^, namely through randomized controlled trials with an active control group^33,34^ and clearly defined training mechanisms^33,35,24^ implemented in a variable, dynamic and adaptive training schedule^18,36,37^ across a large sample of participants and with a comprehensive set of outcome measures taken at multiple timepoints. The present study represents such an approach, following current best practices of the field^33,36^ to interrogate whether a core facet of cognitive control—response inhibition—can be improved and whether this leads to changes in other domains of functioning. We found that each group improved throughout the intervention on their trained process and that training effects remained present up to 1 year after the end of training, suggesting that the training was highly effective at improving the targeted cognitive processes. We also found that the proactive slowing exhibited in the experimental group became manifest as general slowing on other cognitive control tasks. Although it has been shown that training response inhibition can increase proactive control^62^, the absence of reduced errors on cognitive control tasks in the present study suggests that such slowing does not bestow any strategic advantage. The fact that the two training groups improved on the targeted function strengthens the evidence of absent training effects on any far transfer measure or underpinning neurocognitive outcome. Bayesian analyses demonstrate evidence of the absence of transfer effects on any of the tested domains or brain mechanisms implicated in cognitive control. Furthermore, the present study also addresses two recent hypotheses for the large heterogeneity of effects in cognitive training studies. The first of these proposes that the occurrence of far transfer depends on and is, indeed, mediated by the occurrence of near transfer^51^. We did not find evidence to support this claim in the current work. Similarly, it has also been argued that far transfer effects might emerge over time and can, therefore, be detected only by testing again at least 1 year after the end of an intervention^29^. Again, we did not find any evidence for such effects. Finally, we were able to leverage the unique opportunity of COVID-19 as a large-scale and unintended stressor that occurred during the period of our study, allowing us to test a commonly held assumption, namely whether cognitive control training would buffer against the onset of mental health difficulties after a stressor^11,47^. We did not find any evidence of such an effect, and, in fact, we found moderately strong evidence of the absence of an effect of appreciable magnitude. In sum, the present study provides evidence against the possibility of training cognitive control in targeted ways to improve associated domains of functioning, at least as instantiated through a response inhibition intervention.

A fundamental feature of virtually all cognitive control interventions is to attempt to bring about improvements by directly increasing the capacity of the targeted function (that is, extend the number of items held in working memory and accelerate the speed of inhibition or flexibility)^24^. This approach is predicated on the assumption that cognitive control is a limited capacity or resource^63^, with little regard for what might motivate its use. The present study demonstrates that such an approach does not impact children’s behavior or underlying neural architecture, at least not through targeting response inhibition. Indeed, resource accounts of cognitive control, although popular for many years, are being debunked on both theoretical and empirical grounds^64^ and replaced with theories that consider cognitive control as inherently goal-oriented processes^65,66^. A growing body of empirical evidence and computational modeling has shown that cognitive control is assigned a value as a function of subjectively perceived effort and the likely reward or goal priority^65,67,68^. Critically, these insights were successfully leveraged recently in the context of aiming to improve cognitive control. For instance, effort-contingent rewards introduced during cognitive control tasks, by means of objective assessments of effort, led to an increased preference of effort in new tasks, such as difficult problems of arithmetic^69,70^. In conjunction with the present findings that cognitive control cannot be changed through artificially inflating capacity, this raises the possibility that cognitive control could be improved in ways that lead to changes in other domains by targeting motivation and effort expenditure, something that has yet to be tested in developmental populations.

We note some limitations in the current work. Although the overall duration was longer than other recent studies demonstrating far transfer^26,29^, there is a possibility that the present training was insufficient in terms of dose or implementation. Furthermore, our sample came predominantly from above-average SES backgrounds. Although there is still some variability in SES, which, when accounted for, does not alter the results, we acknowledge that our findings may not generalize to other samples and, in fact, that such a training might be efficacious for children coming from lower SES backgrounds (although see ref. ^71^).

In conclusion, we followed best practice recommendations for designing cognitive trainings to test whether cognitive control can be improved in durable ways through training response inhibition and whether this leads to changes in associated domains of functioning in a large sample of children and number of outcome measures. Although trained functions improved in both groups and did so up to 1 year after training, and response inhibition training led to more cautious task responding generally, our training did not lead to changes in children’s behavior or associated neural mechanisms. Given the considerable policy implications of how children can be supported in their development, these findings caution against any further investment in seeking to improve response inhibition specifically and cognitive control more generally through trainings that canonically aim to boost these capacities wholesale.

## Methods

### Participants

A total of 262 typically developing children were recruited for the study (6.03–13.31 years; mean age = 8.97 years; females = 52.84%) from schools within Greater London in the United Kingdom (data collection started in May 2019 and ended in May 2021). Sampling occurred by contacting over 2,000 schools in the Greater London area. Of those schools, 20 ended up participating from a diverse range of boroughs. Information material was disseminated among parents of participating schools, and only those children whose parents/carers had signed them up ended up taking part. Participants were excluded on the basis of formal diagnoses of neurodevelopmental disorders as well as a safety protocol for neuroimaging (for example, metal in the body and claustrophobia). After exclusion of incomplete data, our sample consisted of 235 children (6.03–13.31 years; mean age = 8.97 years; females = 51.91%). The ethnic composition of our sample was as follows: Asian = 14.65%; Black = 3.18%; Mixed/multiple ethnic groups = 17.20%; White = 64.33%; Other = 0.63%. SES was assessed based on employment and education of both parents^72–74^ (Supplementary Table 1). There was a positive skew in SES (mean = 1.64; on a scale of 1–5 where 1 is the highest score attainable). Children were randomly assigned to an experimental group training cognitive control (through inhibition) or to an active control group training response speed (Supplementary Figs. 1 and 2), with groups matched for gender and age, school and class based on mean matching. Matching was performed by an experimenter not involved in testing.   

The University College London (UCL) ethics committee approved this study (protocol number: 12271/001). In accordance with this, written informed consent was obtained from parents, and assent was obtained from children after a description of the study was provided.

### Study design

This study had four main phases. After an initial baseline data collection phase at pre-test, the 8-week computerized intervention was administered. This was followed by a post-test and, finally, a 1-year follow-up. Behavioral, questionnaire and neural data (that is, at pre-test, post-test and 1-year follow-up) were collected to examine independent near transfer and far transfer changes. Due to disruptions to in-person testing during the COVID-19 pandemic, no magnetic resonance imaging (MRI) was obtained at 1-year follow-up. Retention was 71.24% from pre-test to post-test and 99.40% from post-test to 1-year follow-up.

#### Training games

Training was programmed on Gorilla Experiment Builder (https://gorilla.sc/), a platform for running behavioral research online. Training was presented in the form of a computerized web-based Treasure Game. The training was designed to last 8 weeks, with four recommended sessions per week, one taking place at school and three at home. Each session was programmed to take approximately 15 min.

Both groups received identical training in terms of narrative, stimuli and intensity (Supplementary Fig. 2). The only difference between the groups was how participants were instructed to respond to the stop stimuli (that is, inhibit for the Experimental Group and respond for the Control Group; further details are provided in the Supplementary Information). Once every week, questions regarding children’s motivation were administered (Supplementary Information).

##### Experimental Group: response inhibition training

To train response inhibition, a stop-signal response task was used. Participants were instructed to press the spacebar on presentation of a ‘go’ signal. On stop trials where a ‘stop’ signal appeared after the ‘go’ signal, participants were instructed to inhibit pressing the spacebar (however, see Supplementary Information Table 4 for specific descriptions of each training game and training mechanism). ‘Go’ and ‘stop’ signal stimuli and inhibition mechanism varied according to the game being played. The stop signal delay (SSD) was initially set at 200 ms. After successful inhibition, the SSD would decrease by 50 ms, and, after failed inhibition, it would increase by 50 ms^75,76^. This ensured that the training was adaptive. Stop trials occurred 26–47% for each training session. To ensure adaptiveness across training sessions, the SSD of each subsequent session was taken from the final ‘stop’ trial of the preceding session on that specific training game.

##### Control Group: response speed training

The response speed training was identical to the experimental condition in all aspects except that a response was required for all signals. Participants were instructed to press the spacebar as quickly as possible. To ensure that training was adaptive for this group, participants had to respond within a time window that was set based on a rolling average of the response time of the previous 10 trials plus two standard deviations. This ensured that the training was adaptive while minimizing the effect of outliers on the response threshold.

### Pre–post tasks

Before and after the training, three assessment timepoints took place onsite at the author’s laboratory: before the training (T0), after the training (T1) and 1-year follow-up (T2). Note that, due to the outbreak of the COVID-19 pandemic in March 2020, some participants completed one or more assessment timepoints online from home. The assessment battery included several child-friendly tasks measuring cognitive control and neural measurements as well as creativity, mental health and academic performance (Supplementary Fig. 1).

### Cognitive control tasks

A total of nine cognitive control tasks were administered, assessing different functions (that is, inhibition, shifting and working memory). For all tasks, participants were presented with practice trials, before main trials were administered, where they had to attain a criterion threshold for accuracy. Additionally, comprehension questions were employed to ensure participants understood the rules for each task (for example, ‘What button should you press if you see a bear on the screen?’). Rules were re-explained if participants answered incorrectly on any of the questions. The experimenter noted if the participant still failed to comprehend the task. All participants managed to pass these comprehension questions; therefore, no individual was excluded from the analysis. The task was presented using Presentation software (https://www.neurobs.com/, version 23). For remote testing during COVID-19, a subset of executive function tasks was administered online via Gorilla (https://gorilla.sc/)^77,78^.

#### Inhibition tasks

##### SSRT task

A measure of cognitive control was administered via a child-friendly version of the SSRT^79^. Ten practice trials were administered before 80 trials of the main task. Each trial started with the presentation of a fixation cross of 1,250 ms. During the task, participants were asked to press the left arrow key when seeing the ‘go’ signal (that is, a honey pot) on the left side of the screen and the down arrow key when the signal appeared on the right side. On 25% of the trials (that is, a ‘stop’ trial), a picture of bees was presented after the honey pot. This served as the ‘stop’ signal. The SSD started at 200 ms, decreased by 50 ms after a successful ‘stop’ trial and increased by 50 ms after an unsuccessful ‘stop’ trial. As a measure of inhibition, a mean SSRT (ms) was calculated using the integration method^80^. Several studies validated the SSRT as a measure of response inhibition^81^, and it is correlated with self-report measures of impulsive behaviors in young adults^75^.

##### Flanker inhibition

The participants completed a child-friendly version of the Eriksen flanker inhibition task^82^. Children were presented with a row of fish on the screen. They were required to focus on the fish in the center (named Chloe) and indicate the direction in which it was swimming (that is, left key response required when the fish was facing left; down key response required when the fish was facing right). Participants were told to ignore the direction that other fish swim in and only indicate the direction that Chloe swam in. On congruent trials, all fish faced the same direction. On incongruent trials, surrounding fish faced the opposite direction to Chloe. Fish were presented for 700 ms before they disappeared. Participants were given a maximum of 2,500 ms to respond from stimulus onset. A total of 20 congruent trials and 20 incongruent trials were administered. This task was chosen because it is a child-friendly task for ages 6 years and up and was validated in several studies^83,84^. The difference in both reaction times and error rates between incongruent trials and congruent trials was calculated separately.

##### Stroop

Participants completed a child-friendly version of the Stroop task^85^. The task was introduced as the ‘Farm Animal’ game, where they were told to match animals to their homes (for example, dog to a kennel). They were presented with both auditory stimuli of an animal sound (for example, ‘bark’, ‘meow’ and ‘croak’ for dog, cat and frog, respectively) and visual stimuli of the animals. Crucially, participants were asked to match animals to where they live (for example, frog to a pond). They were told to listen carefully to an auditory cue indicating the animal type (for example, frog – ‘ribbit’) and not to pay attention to the visual cue of the animal presented on the screen. Trials lasted for 10,000 ms within which participants had to make a response. Although audio stimuli were presented for 600 ms, visual stimuli were presented until participants made a response (maximum of 1,000 ms). A blank screen with a ‘cross’ was presented between trials for 10,000 ms (inter-trial interval (ITI)). On congruent trials, both auditory and visual cues matched (for example, frog presented on screen and ‘ribbit’ tone played). On incongruent trials, auditory and visual cues did not match (that is, dog presented on screen and ‘ribbit’ tone played). Participants completed 72 trials in total, with 36 congruent and 36 incongruent trials. The differences in both reaction times and error rates between incongruent trials and congruent trials were calculated separately.

#### Memory tasks

##### *N*-back

Both the 1-back and 2-back tasks were administered to measure working memory^86^. The task was adapted to be child-friendly and introduced as the ‘Dino-Donut’ game, where participants were told that dinosaurs were lining up to eat some donuts. For the 1-back task, they were told to stop dinosaurs that tried to eat a donut twice in a row and to press the spacebar if they appeared consecutively to stop them. For the 2-back task, they were told that the dinosaurs became sneakier, and this time they should press the spacebar if the same dinosaur appeared two trials prior. Stimuli were shown for 500 ms followed by a 1,500-ms inter-stimulus-interval (ISI). Responses had to be made before the onset of the next stimulus presentation. Participants completed 80 trials in total, 40 for each *N*-back condition. As a measure of error rate, false alarm rate was calculated for both 1-back and 2-back tasks. Reaction times to make a correct response were also calculated.

##### Corsi block-tapping task

Working memory span was assessed using the Corsi block-tapping task, which measures visuo-spatial working memory span with a higher value indicating a higher working memory span^87^. This task consisted of ‘Freddy the frog’ jumping between nine potential locations designed as lily pads. The participants followed the jumps by clicking on the lily pads in a forward sequence. Trials commenced with a countdown from 3 to 1 to alert participants to the start of a trial. Then, the stimulus of the frog jumping was shown for 600 ms for every jump. The ISI was fixed to 600 ms. Participants completed three practice trials with feedback, and there was a total of 14 main trials. Initially, participants had to remember and click on two lily pads. The task employed an adaptive staircase design where the working memory load (that is, number of lily pads to remember) increased by one when participants made two consecutive correct answers. The maximum working memory load attained was used as a working memory span measure.

#### Shifting tasks

##### Cognitive flexibility

A child-friendly version of the cognitive flexibility task assessed participants’ ability for rule switching across dimensions (using sound cues: ‘animal’ or ‘size’). If a sound cue of ‘animal’ was played, participants had to indicate if the animal was a cat or a dog. If a sound cue of ‘size’ was played, participants had to indicate if the animal was big or small^88^. Participants had 10 s to respond, during which the stimuli remained on the screen before the trial timed out. Responses made before 200 ms after stimulus onset were not recorded. The ITI was jittered and ranged from 1,000 ms to 1,200 ms. Stay trials were preceded by a trial with the same rule (for example, deciding on the type of animal was presented twice in a row). During switch trials, the current trial was preceded by a trial in a different dimension (that is, participants had to first respond to the size of the animal and then to the type of animal that is presented). After a practice block, participants completed 40 trials (consisting of 28 stay trials and 12 switch trials). Participants completed 20 single-dimension trials in two blocks and 40 mixed trials in one block. The difference in both reaction times and error rates between switch trials and stay trials was calculated.

##### Flanker shifting

The participants completed a child-friendly version of the Eriksen flanker shifting task^88^. Children were presented with a row of fish on the screen. They were told that all the fish swim in the same direction. However, two colors of fish would appear: orange and purple fish. When orange fish were presented, they were instructed to indicate the direction in which the fish swam (that is, left key response required when the fish faced left; down key response required when the fish faced right). When purple fish were presented, they were instructed to indicate the opposite direction in which the fish swam (that is, left key response required when the fish was facing right; down key response required when the fish was facing left). Fish were presented for 700 ms before they disappeared. Participants were given a maximum of 2,500 ms to respond from stimulus onset. Stay trials were defined as those where the rule for the previous trial was the same as the current trial (that is, purple trial after a purple trial; orange trial after an orange trial). Switch trials were defined as those where a rule change has occurred (that is, purple trial after an orange trial; orange trial after a purple trial). Based on this, there were 28 stay trials and 12 switch trials. The difference in both reaction times and error rates between switch trials and stay trials was calculated.

#### Complex cognitive control tasks

##### AX-CPT

Reactive and proactive control were measured using a child-friendly version of the AX-CPT paradigm^89^. The task was introduced as the ‘Fruit Island’ game. An ‘A’ or ‘B’ cue (that is, dog or cat) was presented in the middle of the screen for 500 ms, followed by an ISI of 750 ms and then a probe ‘X’ or ‘Y’ (that is, orange or apple) during which participants had to make their response. Participants were instructed to press the left key whenever an ‘X’ followed an ‘A’ (that is, AX trials) and to press the down arrow key for all other cue–probe combinations. Importantly, they were instructed to only respond once the probe had been presented and were alerted of this if they made a response before the probe was presented. Participants had a maximum of 6,000 ms to make a response. Responses were followed by an ITI of 1,500 ms. The proportions of the trial types were based on previous studies^89,90^ where 40% of trials were AX trials. All other trials (that is, AY, BX and BY trials) were presented 20% each. Trials were presented randomly. Ten practice trials were administered where feedback was provided, followed by 60 main trials. Proactive Behavioral Index (PBI) was calculated for error rates and reaction times separately^91^.

### Decision-making tasks

Participants were told that they would be playing a series of games where they could win monetary units (MUs) and exchange these for gifts at the end of the experiment. Participants were told that the more MUs they had at the end of all the games, the larger their gift would be. The reward was described in this abstract way to appeal to children of all ages and was previously found to be sufficiently motivating for children of this age and equally so across the age range^92,93^.

#### DG

Participants were allocated six MUs, visually represented in the task as coins on a computer screen. In the offline sample, two boxes were presented, one for the child and one for their ‘partner’. Children were told that they were playing with another child from a different school; in reality, there was no other participant. They were instructed to first click on the MU and then the boxes to divide them, and they were informed that once they had put an MU in a box, they could not change their decision. Counters at the side of the boxes kept track of the number of MUs in either box. During the task, the instructor explicitly informed the participant that they would turn away and not look at the screen. There was no response time limit. The DG measures pro-social decision-making as indicated by how many MUs a participant decides to give to another unknown child. In the online version, children determined their chosen distribution by moving a slider. In this sense, the online task required just one move to distribute the MUs. As in the offline version, children were told that they were playing with another child from another school whom they did not know, when, in reality, there was no other participant. Unlike in the offline sample, however, children could change their minds about their preferred distributions indefinitely and submit their final decision by pressing the spacebars on their computers. Parents were instructed to be present in the room while testing, engaged in an activity such as reading a book and not to influence their children’s participation.

#### UG

The UG consisted of the responder role. Children could accept or reject a single offer of an unfair distribution (1/6) of MUs made by another unknown child in the study. If they rejected the offer, the participant and the unknown other child who made the offer (a computer, in reality) would receive zero MUs. For this game, there was, again, no response limit for the participants.

#### Intertemporal choice task

Intertemporal decision-making was assessed using an intertemporal choice task. In the intertemporal choice task, participants made choices between immediate and delayed reward options. This task measured the extent to which participants discount rewards as a function of how delayed they are via their choices. Participants completed 18 trials (in a fixed order) where they were always presented with a choice between either an immediate or a delayed option. The unit of delay used was days, where every moon depicted indicated one additional day of waiting before the participant would receive their reward. The reward for the delayed option was always eight MUs, and the immediate reward option ranged among two, four and six MUs. For every immediate reward option, participants’ discounting was measured by calculating the percentage of total delayed choices.

### Academic performance

Academic performance scores were collected retrospectively from schools in the form of English and Maths age-standardized scores. Depending on school, English tests included Progress in Reading Assessment, Progress Test in English, Suffolk Reading Test and/or New Group Reading Test, and Maths tests included Progress in Understanding Maths Assessment and/or Progress Test in Maths. As we did not have discipline-specific hypotheses, the main measure for overall academic performance was a composite age-standardized score computed for each participant as the average across all available English or Maths age-standardized scores for that participant; if participants had scores for one test or discipline only, that score was used as a measure of overall academic performance.

### Creativity

Creativity was measured using the Torrence Test of Creative Thinking (TTCT)^94^. The TTCT is the most widely used test of creativity^95–97^. The TTCT consists of verbal and figural versions. In the present study, we used TTCT-Figural form A. Participants were provided with a pencil, an eraser and a printed Torrance activity sheet. Following a protocol, participants were instructed to use 10 min to complete the given stimuli with unique answers and to come up with interesting titles that described their drawings. In case participants finished in less than 10 min, they were encouraged to use the remaining time to add to their answers. It has high test–retest reliability and can predict creativity success^98^.

### Fluid intelligence

Fluid intelligence was measured using WASI-II (ref. ^99^). The WASI consists of two parts: Matrix Reasoning and Vocabulary. WASI Matrix Reasoning measures non-verbal ability, which correlates well with fluid and visual intelligence, and the WASI verbal subtest measures verbal ability, which correlates well with verbal IQ and crystalized intelligence. For Matrix Reasoning, participants were provided with 30 visually depicted incomplete matrices and asked to choose one from the five options that logically follows the missing matrices. For the vocabulary part, participants were presented with 28 words, one at a time, and asked to verbally define or describe the word presented. WASI-II has high reliability and validity^100^ and provides a good estimate of intelligence.

### Mental health

#### SDQ

The parent-report version of the SDQ^101^ was used to measure internalizing and externalizing difficulties. The SDQ is a 25-item scale consisting of five subscales (emotional problems, conduct problems, peer relationship problems, prosocial behavior and hyperactivity/inattention), each of which includes five questions. Parents rate their child’s behavior over the previous 6 months. Each question has the following response options: 0 = not true, 1 = somewhat true and 2 = certainly true. For each scale, the responses can be summed to provide a total score for that scale. In non-clinical samples, such as are included in the present study, it has been recommended to combine the scales into two further subscales representing ‘internalizing’ and ‘externalizing’ problems^102^. The internalizing subscale is calculated by summing the emotional problems and peer relationship problems subscales, and the externalizing subscale is calculated by summing the hyperactivity/inattention and conduct problems subscales. We, therefore, used this approach in the present study. The SDQ has high validity and reliability^103^.

#### Child and Adolescent Symptom Inventory-4R

The Child and Adolescent Symptom Inventory-4R (CASI-4R)^104^ is a parent-report rating scale that evaluates behaviors related to the disorders that are included in the *Diagnostic and Statistical Manual of Mental Disorders* in young people aged 5–18 years. In the present study, the CASI-4R subscales relating to attention-deficit/hyperactivity disorder (ADHD), generalized anxiety disorder, major depressive episode, depressive disorder, conduct disorder, social phobia and separation anxiety were included. Parents were asked to rate their child’s overall behavior. Each question has the following response options: 0 = never, 1 = sometimes, 2 = often and 3 = very often. Previous studies found that the CASI-4R has good test–retest reliability, validity and internal consistency^105^.

### Apathy Evaluation Scale, informant version

The Apathy Evaluation Scale, informant version (AES-I), was used to assess apathy^106^. The AES-I includes 18 items relating to cognitive, behavioral and emotional apathy. We asked parents to rate their child’s behavior over the previous 4 weeks. Each question is rated on a four-point scale (not at all, slightly true, somewhat true and very true), with higher scores reflecting greater apathy.

### MRI measures

MRI data were acquired with a standard whole-head coil on a 3.0-Tesla Siemens Prisma scanner at the Birkbeck-UCL Centre for Neuroimaging. To limit head motion, participants were asked to keep their heads as still as possible. Foam inserts were used between the head and the head coil to ensure a snug fit. Visual stimuli were projected onto a screen in the magnet bore that could be viewed via a mirror attached to the head coil. During the acquisition of the structural and diffusion tensor imaging (DTI) scan, participants watched cartoons without sound.

#### Task-related functional MRI

The same SSRT task used outside of the scanner was employed where two runs (54 trials each, jittered ITI = 2,200–3,000 ms) were administered. Each run lasted approximately 5 min each and was acquired using T2-weighted echo planar imaging (EPI; TR = 1.25 s, TE = 35.2 ms, sequential acquisition, 60 slices of 2 × 2 × 2 mm^3^ voxels, field of view 1,696 × 1,696, 106 × 106 matrix, in-plane resolution = 2 mm). The task was presented using Presentation software (version 23).

#### Cortical thickness

High-resolution T1-weighted images were acquired using a magnetization-prepared rapid gradient echo sequence (MP-RAGE; TR = 2.30 s TE = 2.98 ms, flip angle = 8°, slices = 1 × 1 × 1 mm^3^ voxels, field of view 256 × 256). A total of 208 slices per participant (voxel size = 1 × 1 × 1 mm^3^) were collected, and the acquisition matrix ranged 256 × 256.

#### Resting state

Participants completed one run lasting 5 min (212 EPI volumes, 60 slices per volume, voxel size 2 × 2 × 2 mm^3^, TR = 1,250 ms, TE = 35.2 ms, flip angle = 65°). Participants were instructed to observe a fixation cross presented on a screen. For spatial normalization and anatomical localization, a structural scan was obtained (see ‘Cortical thickness’ subsection above). Finally, to improve functional-to-anatomical co-registration, a field map scan was acquired (one EPI volume, 72 slices per volume, voxel size 2 × 2 × 2 mm^3^, TR = 8,000 ms, TE = 66 ms, flip angle = 90°).

#### DTI

Diffusion imaging was acquired while children were awake. A total of 72 contiguous near-axial slices were acquired for each volume, using an acquisition sequence fully optimized for clinical tractography, providing isotropic (2 × 2 × 2 mm) resolution and whole head coverage (matrix size 104 × 104 × 72, TR = 3,600 ms, TE = 92 ms). Then, 100 diffusion-weighted volumes (50 × b-value of 1,000 s mm^−^^2^, 50 × b-value of 2,000 s mm^−^^2^) and five volumes without diffusion gradient were acquired.

### Pre-processing and statistical analysis

Outliers were removed for all measures. Datapoints falling two standard deviations below or above the mean were excluded.

#### Cognitive control factors

Outliers were removed from each cognitive control measure. Datapoints falling two standard deviations below or above the mean were excluded. Then, a confirmatory factor analysis (CFA) was performed using ‘lavaan’ in RStudio to create latent factors of executive functions^107^. For T0 data, multiple models were fit; however, the model failed to converge for most models, with some of them displaying negative variances, suggesting that models were mis-specified. Only two models converged: a model with a single factor encompassing all tasks and a model with three subfactors of inhibition, shifting and memory. There were no significant differences in model fits (Δ*χ*^2^ (3) = 1.69, *P* = 0.638). The inhibition factor was extracted to examine correlations at T0 with the other domains. To examine training-related changes in executive functions, the factor analysis was conducted separately for error rate and reaction time data. This was done because a factor solution could not be found when composite measures of error rates and reaction times were used. For the error rate factor specifically, inclusion of flanker inhibition indices caused non-convergence of models and was excluded from analysis. Based on previous literature, factor loadings were constrained by timepoints to allow for pre–post comparisons establishing weak factorial invariance^26^. Values for each individual were extracted from this for further analysis. This was done separately for error rates and reaction times, where a larger value indicated a larger error rate or reaction time.

#### Creativity

The TTCT responses were scored according to the Streamlined Scoring Guideline^108^. The responses were scored with respect to five norm-based creativity measures: fluency, originality, abstractness of titles, elaboration and resistance to premature closure. A higher score in any of the five subcategories indicates more unique answers and higher levels of creativity. In the present study, all responses were scored by a single scorer, and a sum score of all five categories was used for the analyses. To establish consistency, the scorer scored a random sample of 10 responses two times with 2 weeks in between. Eighty-six percent of the scores were consistent across the two separate scorings.

#### Mental health

A CFA was performed using ‘lavaan’ in RStudio to create latent factors of mental health^107^. Based on previous literature, factor loadings were constrained by timepoints to allow for pre–post comparisons establishing weak factorial invariance^26^. Factors of externalizing problems and internalizing problems were created. Specifically, externalizing problems from the SDQ and CASI-ADHD problems loaded on the externalizing factor. Internalizing problems from the SDQ, CASI-social phobia, CASI-separation anxiety and CASI-depression loaded on the internalizing factor. Values for each individual were extracted from this for further analysis, where a larger value indicated greater mental health problems.

#### MRI measures

##### Task-related functional MRI

Each individual’s functional scans were realigned to correct for head motion by initial realignment to first image and second realignment to mean image. The realigned scans were co-registered with anatomical T1-weighted images and spatially normalized to the standard Montreal Neurological Institute (MNI) space by resampling to a voxel size of 2 × 2 × 2 mm^3^. Normalized images were smoothed with an 8-mm Gaussian filter. Fixed statistical effects were calculated at the individual level by modeling each trial condition (‘stop’ successful, ‘stop’ unsuccessful, ‘go’ successful and ‘go’ unsuccessful) with a box car function convolved with the canonical hemodynamic response function. To reduce movement-related artifacts, six motion parameters were included as regressors as well as an additional regressor to model images that were corrupted due to head motion of more than 1.5 mm and were replaced by interpolations of adjacent images (<10% of participant’s data). To examine training-related changes from pre-test to post-test in ‘stop’ versus ‘go’ trial condition, the Sandwich Estimator Toolbox for Longitudinal and Repeated Measures Data version 2.1.0 was employed (SwE, toolbox for SPM, Guillaume et al.^109^). Repeated-measures ANOVA was conducted at the group level, with the ‘stop’ successful condition and ‘go’ successful condition entered as fixed effects and a subject factor entered as random effects. Family-wise error (FWE) corrections at *P* < 0.05 were applied to the data. Moreover, using the MarsBaR Toolbox^110^ implemented in SPM12, we extracted functional activity from the right IFG selected from the probabilistic Harvard-Oxford atlas^111^ (thresholded at 20%, center of mass: 51, 28, 8). Beta values for each ROI (that is, successful ‘stop’ trials versus successful ‘go’ trials) were extracted for further statistical analyses outside of SPM.

#### Cortical thickness

After converting the DICOM files to NifTI using dcm2niix, structural MRI images were processed with FreeSurfer^112^ (version 6.0.0, http://surfer.nmr.mgh.harvard.edu) to label and segment cortex and white matter. All scans were then visually inspected for quality, and, if necessary, segmentation was manually corrected in FreeSurfer. Four independent inspectors conducted these checks, and one final inspector performed a final inspection of all scans. After corrections, scans were re-segmented using FreeSurfer. If the quality of scans was inadequate, they were excluded from the final analysis. Based on this, data were available from 141 participants. After pre-processing, sulcal and gyral features across individual participants were aligned by morphing each participant’s brain to an average spherical representation that accurately matches cortical thickness measurements across participants while minimizing metric distortion. A 10-mm Gaussian smoothing kernel was applied to the data to reduce measurement noise but preserve the capacity for anatomical localizations^113,114^. Cortical thickness data were analyzed using the SurfStat toolbox for MATLAB^115^ (https://www.math.mcgill.ca/keith/surfstat). Findings from the surface-based analyses were controlled for multiple comparisons using random field theory^5,113,115^. This reduced the chance of reporting an FWE. We ran whole-brain models looking at changes in cortical thickness after training by testing for a Session-by-Group interaction. Using the Desikan–Killiany atlas^116^, cortical thickness was extracted from the right IFG (comprising the right pars triangularis, the pars opercularis and the pars orbitalis) to look at the specific interaction within this region.

#### Resting state

Processing of resting-state functional connectivity (RSFC) data was completed with the ABCD-HCP pipeline (https://github.com/DCAN-Labs/abcd-hcp-pipeline), which is modified from the original HCP pipelines^117^. In brief, this pipeline consists of six stages. First, the PreFreeSurfer stage normalizes anatomical data. This normalization includes brain extraction, denoising and then bias field correction on anatomical T1-weighted and/or T2-weighted data. To improve output image quality, ANTs DenoiseImage attempts to remove scanner noise from T1 and T2 anatomical images by modeling scanner noise as a Rician distribution, and ANTs N4BiasFieldCorrection attempts to improve bias field correction. Second, the FreeSurfer stage constructs cortical surfaces from the normalized anatomical data. This stage also performs surface registration to a standard surface template, and surfaces are refined using the T2-weighted anatomical data. Third, the PostFreeSurfer stage transforms the volumes to a standard volume template space using ANTs nonlinear registration and the surfaces to the standard surface space via spherical registration. Fourth, the fMRIVolume stage performs processing of the functional data, including correction for functional distortions via reverse-phase encoding spin echo images, intensity normalization to a whole-brain-mode value of 1,000, within-run correction for head movement and registration to the standard template. Fifth, the fMRISurface stage maps the normalized functional volumes to the standard surface template. The BOLD functional MRI volumetric data were sampled to each participant’s original mid-thickness left and right hemisphere surfaces constrained by the gray matter ribbon. These surfaces were then combined with volumetric subcortical and cerebellar data into the CIFTI format using Connectome Workbench (https://www.humanconnectome.org/software/connectome-workbench), creating full brain timecourses excluding non-gray matter tissue. The resting-state timecourses were then smoothed with a 2-mm full-width at half-maximum kernel applied to geodesic distances on surface data and Euclidean distances on volumetric data. Finally, the DCANBOLDproc stage performs further denoising steps to reduce variance unlikely to reflect neuronal activity. These denoising steps include a respiratory filter to improve framewise displacement estimates, temporal masks to flag motion-contaminated frames with a filtered framewise displacement greater than 0.3 mm, demeaning, detrending, interpolation across censored frames and a band-pass filter (0.008 Hz < f < 0.1 Hz).

After processing, time series of RSFC data were extracted using the Gordon-333 parcellation^118^, which includes 333 parcels (ROIs) that cover the whole cortical surface. These time series were further motion censored at a framewise displacement greater than 0.2 mm. Then, parcels were grouped for the networks of interest (FPN and CON), and Pearson correlations across parcels within each network were run. We then computed the mean z-score across all correlations within each network. Therefore, we obtained an RSFC value (z-score) for each network of interest, participant and timepoint.

#### DTI

The data were initially visually inspected. Volumes with extreme artifacts or corruption were removed. Across the dataset, the average number of volumes removed was 0.27 (range = 0–5) at T0 and 0.97 (range = 0–10) at T1, accounting for 0.5% of the total number of volumes acquired. Data was then pre-processed using ExploreDTI (https://exploredti.com/). The data were corrected for head motion, eddy current distortions and EPI distortions, and the *B*-matrix was rotated^119^. Remaining outliers due to head motion and cardiac pulsation were excluded using REKINDLE. The tensor model was fitted to the data using a nonlinear least square fitting procedure. DTI scalar maps, including fractional anisotropy and mean diffusivity, were calculated and exported. A whole-brain tractography algorithm using Euler integration and the following settings was applied: step size = 0.5 mm, fractional anisotropy threshold ≥ 0.15 and angle threshold ≤ 35. Whole-brain tractography was exported to TrackVis (https://trackvis.org/) to perform virtual in vivo dissections for the right hemisphere. The connections were dissected in regions corresponding to the putamen and the frontal lobes, providing measures for the fronto-putamen connections. All dissections were completed after ensuring intra-rater reliability. This was tested with the use of 10 participants from the present study, dissected twice by the same dissector. Reliability was tested using a two-way mixed intra-class correlation coefficient (ICC)^120^. For all tracts, the ICC for single measures reached greater than 0.90. For each tract, fractional anisotropy and mean diffusivity were calculated. These measures reflect the structural integrity of the white matter connection and may indicate microstructural differences, such as myelination, axonal integrity and how compact fiber bundles are^121^. Fractional anisotropy is the degree of directionality of water motion within a particular voxel. Mean diffusivity is the average diffusion of water motion within a voxel.

#### Training-related changes

Mixed models were used to examine training-related changes using the ‘lme4’ package in R (version 4.3.1*)*. In this model, the main effects of training group and session were examined as well as the interaction between Group and Session. Age and gender was added into the model as a covariate. Significant interaction effects between Session and Group were interpreted as presence of training-related changes and followed up with post hoc paired *t*-tests. In a subset of available tasks, maintenance of training-related changes was examined between pre-test (T0) and 1-year follow-up (T2). For evidence of null effects, we report the BF in favor of the null model (the model without a Group-by-Session interaction) over the training model (the model with a Group-by-Session interaction) for each measure of interest^122^. The prior is set to be the model with main effects of Group and Session. We isolate this particular interaction as our training effect of interest where BF_10_ < 1 suggested evidence for the null hypothesis (that is, no training-related changes).

#### Data imputation

For all measures (unless specified otherwise), multiple imputations by chained equations (MICE) was used to impute missing data (predictive mean matching; iterations = 20, *n* datasets = 100; Supplementary Fig. 4). A single imputed dataset was used, as this was necessary in conducting mixed models with post hoc tests and factor analysis. We ensured the replicability of these results by re-running the process multiple times and choosing a dataset at random. Missing data were imputed using the MICE package in R (50 datasets created, 50 maximum iterations), and quickpred was used to create the imputation model^123^. Factors of executive function (at T0) and mental health factors were imputed using full information maximum likelihood (FIML) in ‘lavaan’.

#### Correction for multiple comparisons

The Benjamini–Hochberg procedure^55^ was applied to mixed model analysis testing for training-related changes. We used the adjustment method BH with the function p.adjust in R. For the results that are significant, we report their adjusted *P* value after correction to control FDR with multiple testing.

#### Test–retest reliability

To assess the reliability of outcome measures, we looked at both test–retest reliability and split-half reliability. To assess test–retest, ICCs were calculated using the ‘psych’ package in R. ICC(2,1) was chosen to allow different means at different timepoints using a two-way random-effects model. ICC was calculated on all available timepoints for each given measure. Percentage accuracy and mean reaction time for correct response were calculated for each participant per task for intra-class comparison (Supplementary Table 2). The data used for the reliability tests were not imputed. The Spearman–Brown coefficient was calculated using the ‘splithalfr’ package in R^124^. Percentage accuracy and mean reaction time for the first half and second half of the experiments were compared in the executive function tasks to test internal reliability (Supplementary Table 3). Mean percentage delayed choice was tested for Temporal Discounting Task. For questionnaires, Cronbach’s alpha was tested using the ‘psych’ package in R.

### Reporting summary

Further information on research design is available in the Nature Portfolio Reporting Summary linked to this article.

## Online content

Any methods, additional references, Nature Portfolio reporting summaries, source data, extended data, supplementary information, acknowledgements, peer review information; details of author contributions and competing interests; and statements of data and code availability are available at 10.1038/s41593-024-01672-w.

### Supplementary information

Supplementary Information Supplementary Methods and Results, Supplementary Figs. 1 and 2 and Supplementary Tables 1–7.

Reporting Summary

### Source data

Source Data Fig. 1 Statistical source data.

Source Data Fig. 2 Statistical source data.

Source Data Fig. 3 Statistical source data.

Source Data Fig. 4 Statistical source data.

Source Data Fig. 5 Statistical source data.

Source Data Fig. 6 Statistical source data.

## Data Availability

The raw data are available from the corresponding authors upon reasonable request. The processed data necessary to reproduce the central findings of this study are available at our GitHub page: https://github.com/ucjuliy/BIGDEVBRAINTRAIN.git. Source data are provided with this paper.

## Code availability

Custom R scripts were used to analyze and plot all data. Code is available online at our GitHub page: https://github.com/ucjuliy/BIGDEVBRAINTRAIN.git.
