# Supplementary material for: Cognitive control training with domain-general response inhibition does not change children’s brains or behavior
Source: Nat Neurosci. 2024 Jun 4;27(7):1364–75. doi: 10.1038/s41593-024-01672-w (PMC11239524; doi:10.1038/s41593-024-01672-w)
Supplement: Supplementary file 1 — Supplementary Methods and Results, Supplementary Figs. 1 and 2 and Supplementary Tables 1–7. [file 41593_2024_1672_MOESM1_ESM.pdf]

nature neuroscience

Supplementary information https://doi.org/10.1038/s41593-024-01672-w

Cognitive control training with domain-general response inhibition does not change children's brains or behavior

In the format provided by the authors and unedited

Supplementary Materials

Training Protocol

Participants were introduced to the training games as the 'Treasure Game' with the narrative that they had flown a plane, which had to crash land in the desert. In order to fix their plane, they were required to obtain spare parts from a sage, living in a distant cave. To get to the sage, participants had to travel through 4 different worlds (i.e. forest, desert, snow and mountains), after which they had to go back through the same worlds to return to the plane. While travelling through each world, participants could collect coins and gems, which could be used to trade for spare parts with the sage. Gems and coins were collected in the context of seven different games (see Figure 4SM) that were designed to train inhibition (Experimental group) and response speed (Control group). The seven training games were 1) Treasure collect, 2) Mining, 3) Chest picking, 4) Conveyor belt, 5) AB Driving, 6) Hold-and-Release (HR) Driving and 7) Forest Escape (for details on training mechanisms see Table 4SM). Each training session entailed a combination of two games, which was set in a pre-assigned order at the start of training. Before starting the games, participants were presented with an option of three different caves that they could choose from to encourage engagement and a sense of agency.

Session recoding and inclusion. For both groups, sessions were recoded based on date, meaning any data logged on the same date would be grouped in the same session. Since the implementation of the games differed in terms of key presses and mechanisms tested (Table 2SM), we only included sessions for participants that had a minimum of 2 games and, for the experimental group only, sessions that had at least two games with valid SSRT measures (i.e. positive SSRT values). For the control group, reaction times were included that were within 2 standard deviations of the mean reaction time per participant.

Motivation Questionnaire. The questionnaire consisted of 6 items: "1. I like the training; 2. I like to do the training; 3. I do not always feel like training; 4. I think I can become better through

the training; 5. I find the training boring; 6. I am getting better at the training tasks". Items 3 and 5 were reverse coded.

Parental Beliefs

In a subset of our sample, we collected data on parental beliefs (*N* = 33). Specifically, parents were asked which training group they believed their child to be assigned to. There were no differences amongst parental beliefs between the training groups (χ^2 (2) = 2.49, *p* = 0.287). 54.55% of parents reported their child's training group correctly (not significantly different from chance: χ^2 (1) = 0.12, *p* = .728.

Covid-19 Information

We assessed parent report of whether their children had been infected with Covid-19 or suspected their children of being infected of Covid-19 (Table 5SM) as well as perceived stress before and after Covid-19 (Table 6SM). We found that there was no difference between the two groups in actual or suspected cases of Covid-19 (χ^2 = 2.13, *p* = 0.344). We also, did not find a significant interaction between Session and Group in perceived stress (F (1, 138.86) = 0.162, *p* = 0.688, η^2 = 0.001, BF_{10} = 0.184).

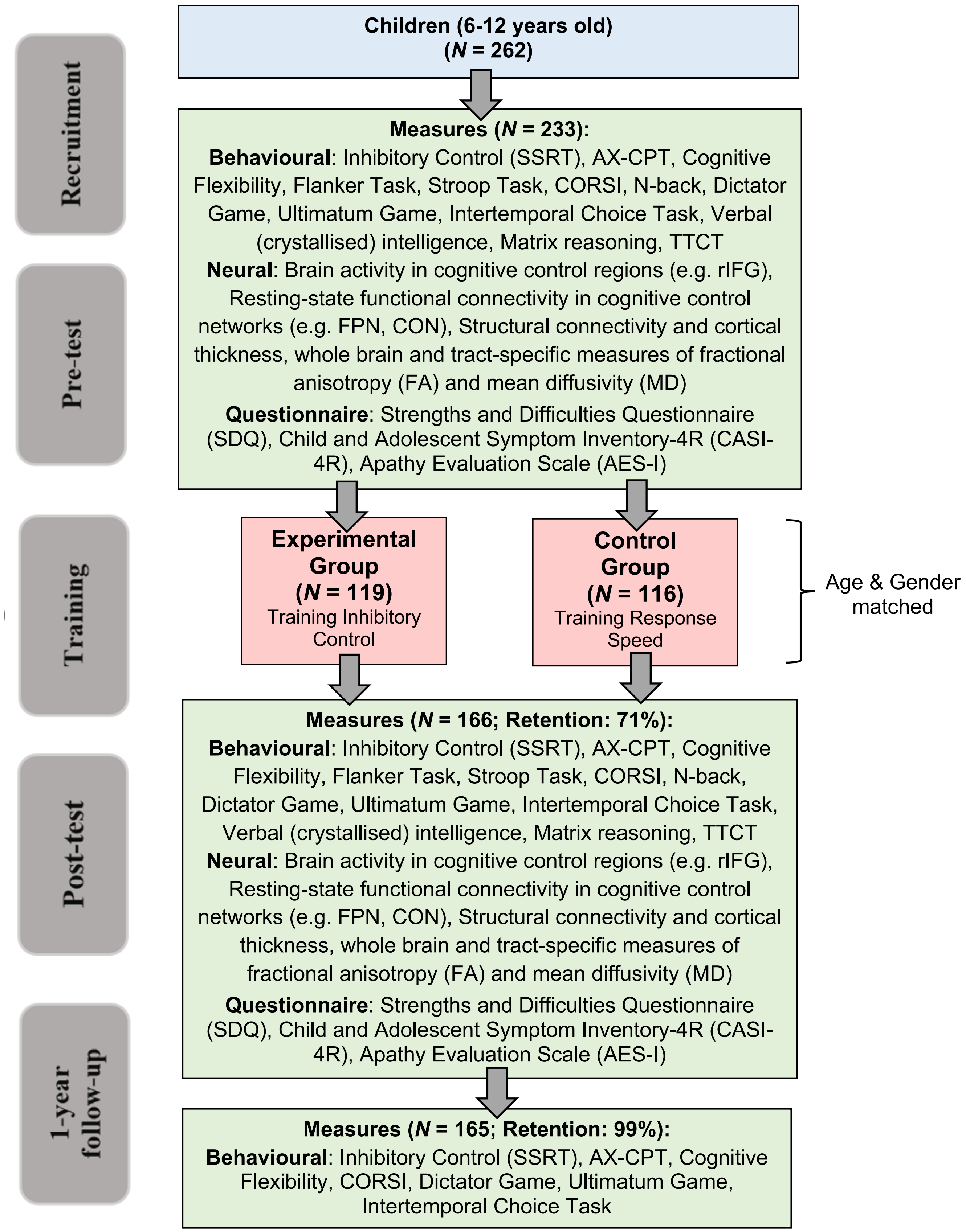

Figure 1SM. Study design. Training design and pre-post as well as 1-year follow-up measurements.

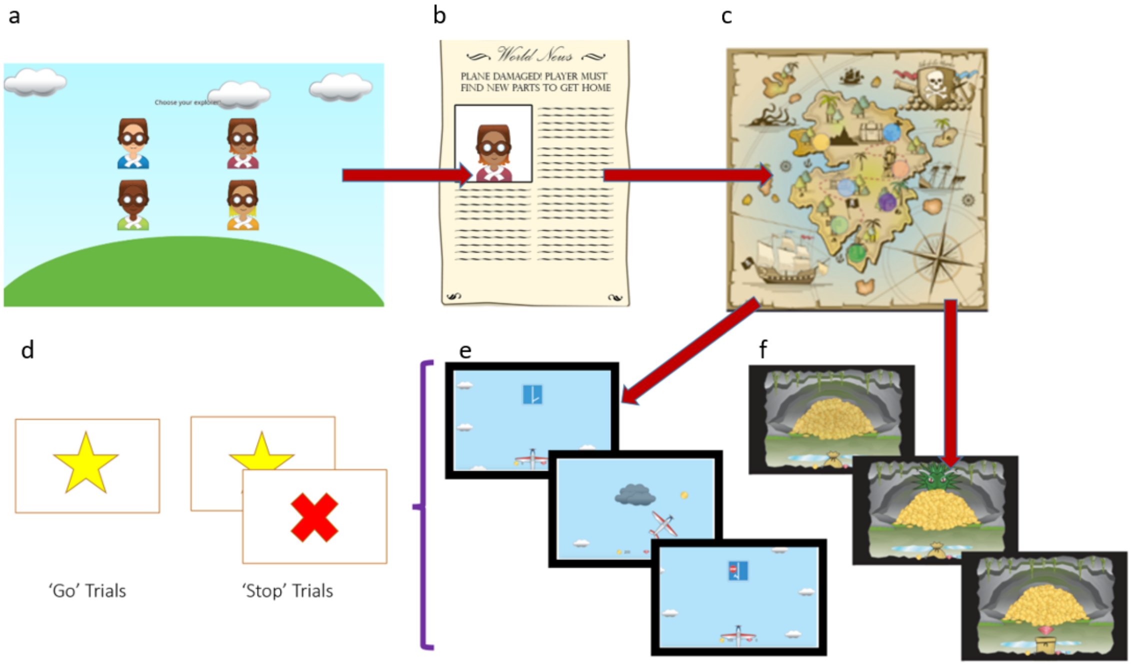

Figure 2SM. Training design. Both groups were administered the same protocol, comprising a variety of adaptive and gamified tasks. **a-c,** Children were told that they were a pilot who had crashed their plane on an island and had to navigate the island to earn coins. **d,** Coins could be earned through games which operated on the same principle where they consisted of 'go' vs 'stop' trials. **e-f**, Examples of these games have been provided.

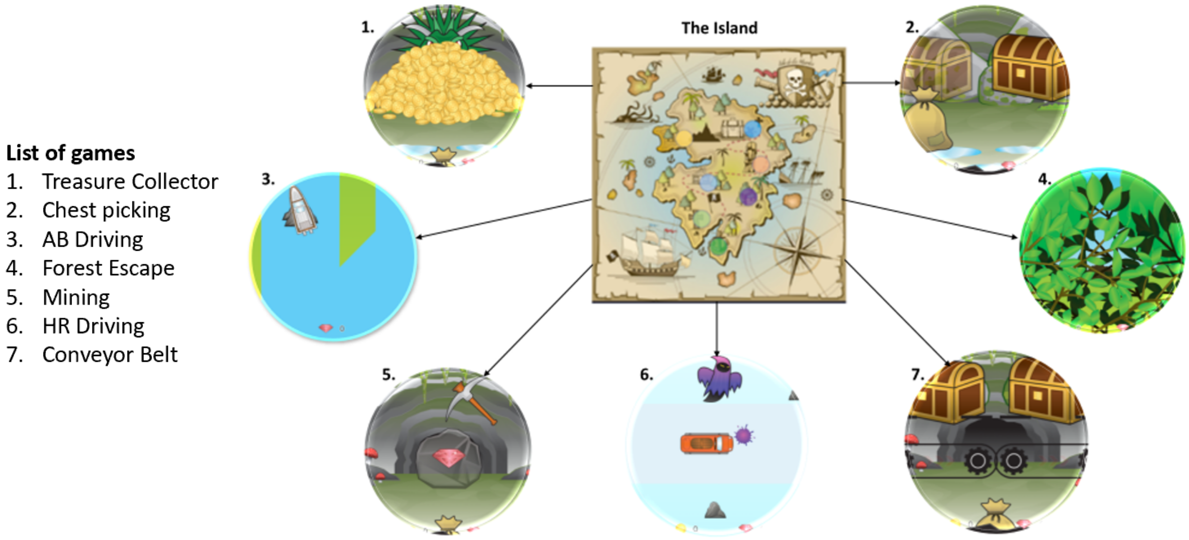

Figure 3SM. Training games. Participants had to navigate an island to find coins. Coins could be earned by playing the different games.

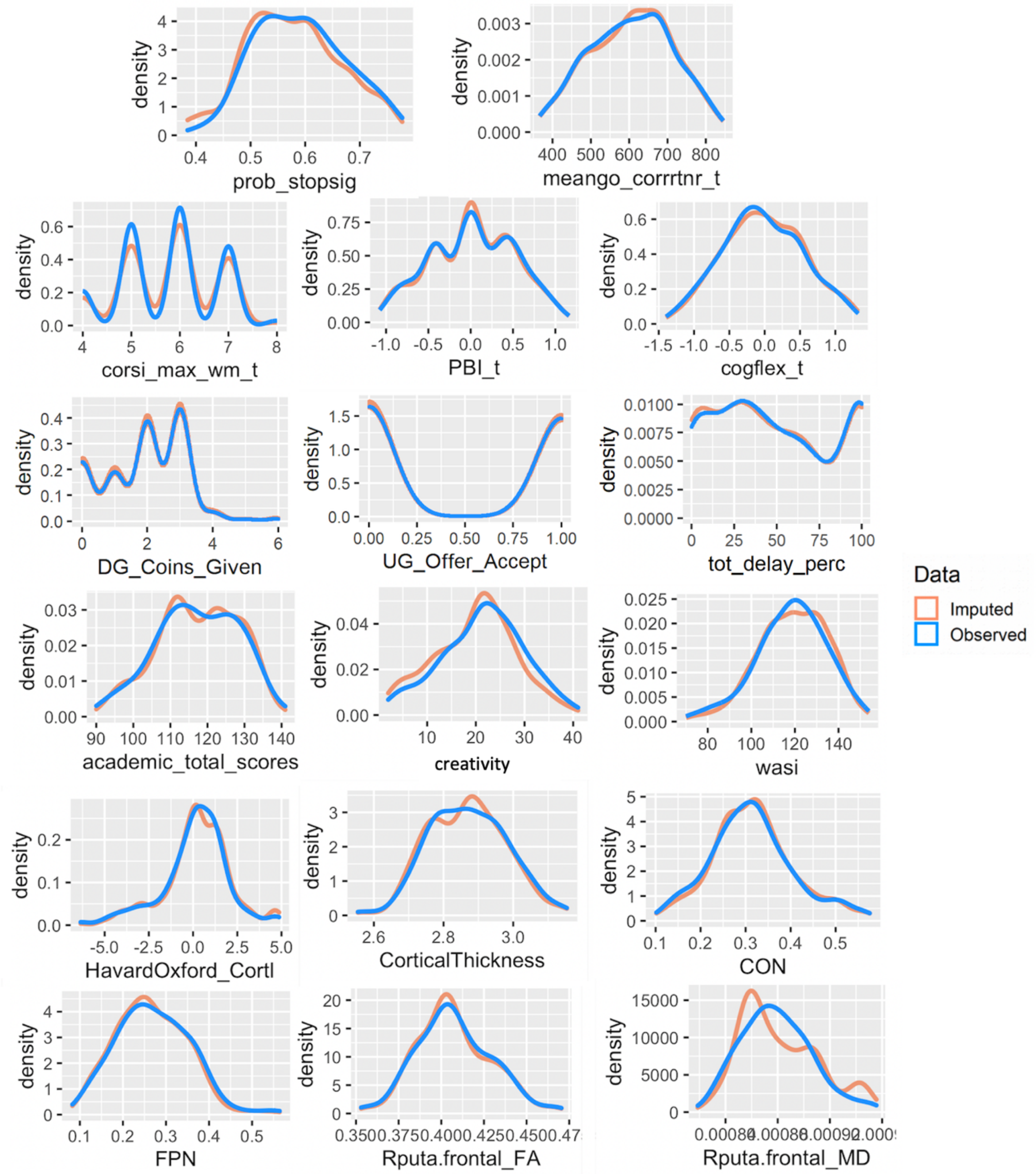

Figure 4SM. Imputation of data. Distributions of imputed vs observed data have been shown for main measures of interest.

Table 1SM. Criteria for assessing socioeconomic status (SES).

Score	Education	Employment
**1**	Postgraduate (MSc, MA, PhD) or professional qualification (e.g. law or accountancy training)	Managerial and professional occupations (e.g. company director, head of HR, lawyer, dentist)
**2**	Undergraduate (BA, BSc) or equivalent (HND/HNC, City and Guilds Qualification, NVQ level 4)	Intermediate occupations (e.g. police officer, administrative assistant, travel consultant)
**3**	A-level, AS-levels, NVQ level 3, BTEC diplomas	Small employers and own account workers (e.g. interior designer, garden designer, baker)
**4**	GCSES, CSEs, O-levels, NVQ levels 1 & 2	Lower supervisory and technical occupations (e.g. interior design assistant, finance officer)
**5**	No formal qualifications	Semi-routine and routine occupations (e.g. yoga teacher, leather crafter, bookseller)

Table 2SM Test-retest reliability (ICC) for Task Variables and Questionnaires

Task Variables	ICC (intraclass correlation coefficients)	*p*	95%CI (lower - upper bound)
**AXCPT accuracy**	0.9	<0.0001	0.87-0.93
**AXCPT RT**	0.74	<0.0001	0.61-0.83
**cogflex accuracy**	0.34	0.0011	0.13-0.52
**cogflex RT**	0.61	<0.0001	0.32-0.77
**Corsi MemorySpan**	0.66	<0.0001	0.48-0.78
**Flanker_inhibition accuracy**	0.96	<0.0001	0.95-0.97
**Flanker_inhibition RT**	0.85	<0.0001	0.80-0.88
**Flanker_shifting accuracy**	0.97	<0.0001	0.96-0.98
**Flanker_shifting RT**	0.92	<0.0001	0.90-0.94
**NBack accuracy**	0.36	0.00037	0.16-0.53
**NBack RT**	0.64	<0.0001	0.50-0.75
**Stroop accuracy**	0.71	<0.0001	0.58-0.80

**Stroop RT**	0.69	<0.0001	0.56-0.79
**DG offer**	0.38	<0.0001	0.26-0.48
**unfair offer acceptance**	0.36	<0.0001	0.25-0.46
**TempDiscounting**	0.59	<0.0001	0.49-0.68
**reading/math**	0.74	<0.0001	0.67-0.79
**WASI**	0.68	<0.0001	0.51-0.78
**creativity**	0.178	0.00012	0.028-0.32
**rIFG activation**	0.22	0.024	0.0027-0.42
**FPN**	0.21	0.0057	0.048-0.36
**CON**	0.23	0.0023	0.073-0.38
**rIFG cortical thickness**	0.71	<0.0001	0.62-0.78
**Rputa.frontal_FA**	0.65	<0.0001	0.54-0.73
**Rputa.frontal_MD**	0.72	<0.0001	0.63-0.79
**AES**	0.64	<0.001	0.58 - 0.70
**SDQ**	0.76	<0.001	0.71 - 0.80
**SDQ internalising**	0.68	<0.001	0.62 - 0.73
**SDQ externalising**	0.75	<0.001	0.70 - 0.79
**CASI**	0.77	<0.001	0.73 - 0.81
**CASI adhd**	0.79	<0.001	0.74 - 0.82
**CASI mdd**	0.53	<0.001	0.46 - 0.60
**CASI sepanx**	0.66	<0.001	0.60 - 0.71
**CASI socphob**	0.73	<0.001	0.67 - 0.77

Intraclass correlation coefficient (ICC) was tested on all available timepoints for each given measure to examine test-retest variability. ICC(2,1) was chosen to allow different means at different timepoints using two-way random-effects model.

Table 3SM Split-half reliability (Spearsman-Brown Coefficient) for Task Variables and Questionnaires

Task Variables	SB_coefficient_T0	SB_coefficient_T1	SB_coefficient_T2

**AXCPT accuracy**	0.567	0.635	0.719
**AXCPT RT**	0.929	0.893	0.915
**cogflex accuracy**	0.659	0.735	0.465
**cogflex RT**	0.833	0.913	0.899
**Flanker_inhibition accuracy**	0.816	0.625	NA
**Flanker_inhibition RT**	0.951	0.915	NA
**Flanker_shifting accuracy**	0.72	0.713	NA
**Flanker_shifting RT**	0.95	0.918	NA
**NBack accuracy**	0.494	0.481	NA
**Nback RT**	0.772	0.763	NA
**Stroop accuracy**	0.776	0.748	NA
**Stroop RT**	0.86	0.783	NA
**AES**	0.86	0.87	0.87
**SDQ**	0.7	0.7	0.69
**SDQ internalising**	0.65	0.66	0.66
**SDQ externalising**	0.75	0.78	0.77
**CASI adhd**	0.92	0.94	0.93
**CASI mdd**	0.74	0.81	0.81
**CASI sepanx**	0.86	0.78	0.79
**CASI socphob**	0.83	0.84	0.86

Spearman-Brown coefficient was calculated for first half and second half of the experiments were compared in the executive function tasks to test internal reliability. Cronbach's alpha was tested for questionnaires.

Table 4SM. Table of stimulus-response instructions for each game, across the different training groups.

Game	Stimulus	Experimental Group (Response inhibition)	Control Group (Response Speed)
	1) Treasure	Press space (go)	Press space

Treasure Collector	2) Dragon	Do not press space (stop)	Press space
Mining	1) Rock<br>2) Gem	Press space (go)<br>Do not press space (stop)	Press space<br>Press space
Chest picking	1) Wobbling treasure chest on the other side to your bag<br>2) Wobbling treasure chest on the same side as your bag<br>3) Dragon	Press space to move to other side (go)<br>Do not press space (stop)<br>Press space to move away from the dragon (go). Do not press space if the dragon is on the other side (stop).	Press space to move to other side<br>Do not press space<br>Press space to move underneath the dragon
Conveyor belt	1) Wobbling treasure chest<br>2) Dragon	Press space to change direction of the belt so that the treasure chest moves towards the bag (go).<br>Avoid the chest with the dragon behind it by pressing space to change the direction (stop).	Press space to change direction of the belt so that the treasure chest moves towards the bag.<br>Move the chest with dragon behind it by pressing space to change the direction.
AB Driving	1) Sign pointing left or right<br>2) Stop sign	'Left' or 'Right' arrow key (go)<br>Do not press 'left' or 'right' arrow key (stop)	'Left' or 'Right' arrow key<br>Press 'left' or 'right' arrow key
HR Driving	1) Ghost looking at the front of your car<br>2) Ghost looking at back of your car	Take finger off spacebar (go)<br>Keep finger on spacebar (stop)	Take finger off spacebar<br>Take finger off spacebar
Forest Escape	1) Pile of coins<br>2) Monster	Press space bar<br>Do not press space bar	Press space bar<br>Press space bar

Note: Participants allocated to the response speed group make a 'go' response regardless of cue

Table 5SM. COVID information for both training groups

Training Group	Confirmed had COVID	Suspected had COVID	Never had COVID
**Experimental Group**	*6*	*13*	*67*
**Control Group**	*8*	*20*	*60*
Total	*14*	*33*	*127*

Table 6SM. Perceived stress, means by group and session

Training Group	Perceived stress mean (sd)	
	Pre-COVID	Post-COVID
**Experimental Group**	*19.514 (6.875)*	*19.954 (6.653)*
**Control Group**	*17.563 (6.516)*	*17.609 (6.262)*
Total	*18.500 (6.740)*	*18.746 (6.537)*

Table 7SM. Whole-brain analysis for successful vs unsuccessful stops from task-based functional imaging data

Timepoint	Area	Coordinates			p-value
		x	y	z	
**T0**	Right insula	36	22	4	.014
	Left insula	-32	24	0	.025
	Right frontal eye fields	6	20	44	.028
**Training-related changes**	-	-	-	-	-

Repeated measures ANOVA was conducted at the group level, with the stop successful condition and go successful condition entered as fixed effects, and a subject factor entered as random effects. Family wise error-corrections (FWE) at $p < .05$ were applied to the data. No significant areas were observed in training-related changes.
