# Supplementary material for: Cognitive control training with domain-general response inhibition does not change children’s brains or behavior
Source: Nat Neurosci. 2024 Jun 4;27(7):1364–75. doi: 10.1038/s41593-024-01672-w (PMC11239524; doi:10.1038/s41593-024-01672-w)
Supplement: Supplementary file 2 — Reporting Summary [file 41593_2024_1672_MOESM2_ESM.pdf]

nature portfolio

Corresponding author(s): Nikolaus Steinbeis

Last updated by author(s): Aug 8, 2023

Reporting Summary

Nature Portfolio wishes to improve the reproducibility of the work that we publish. This form provides structure for consistency and transparency in reporting. For further information on Nature Portfolio policies, see our Editorial Policies and the Editorial Policy Checklist.

Statistics

For all statistical analyses, confirm that the following items are present in the figure legend, table legend, main text, or Methods section.

n/a	Confirmed	
☐	☒	The exact sample size (*n*) for each experimental group/condition, given as a discrete number and unit of measurement
☐	☒	A statement on whether measurements were taken from distinct samples or whether the same sample was measured repeatedly
☐	☒	The statistical test(s) used AND whether they are one- or two-sided<br>*Only common tests should be described solely by name; describe more complex techniques in the Methods section.*
☐	☒	A description of all covariates tested
☐	☒	A description of any assumptions or corrections, such as tests of normality and adjustment for multiple comparisons
☐	☒	A full description of the statistical parameters including central tendency (e.g. means) or other basic estimates (e.g. regression coefficient) AND variation (e.g. standard deviation) or associated estimates of uncertainty (e.g. confidence intervals)
☐	☒	For null hypothesis testing, the test statistic (e.g. *F*, *t*, *r*) with confidence intervals, effect sizes, degrees of freedom and *P* value noted<br>*Give P values as exact values whenever suitable.*
☐	☒	For Bayesian analysis, information on the choice of priors and Markov chain Monte Carlo settings
☒	☐	For hierarchical and complex designs, identification of the appropriate level for tests and full reporting of outcomes
☐	☒	Estimates of effect sizes (e.g. Cohen's *d*, Pearson's *r*), indicating how they were calculated

Our web collection on statistics for biologists contains articles on many of the points above.

Software and code

Policy information about availability of computer code

Data collection	Presentation (www.neurobs.com; Version 23); Gorilla (www.gorilla.sc)
Data analysis	SPM12, the Sandwich Estimator Toolbox v2.1.0 (Guillaume et al., 2014), the MarsBaR Toolbox (Brett et al. 2002), Dcm2niix (V1), FreeSurfer (Version 6.0.0), Matlab (2021a), SurfStat (https://www.math.mcgill.ca/keith/surfstat), ABCD-HCP pipeline (https://github.com/DCAN-Labs/abcd-hcp-pipeline), Connectome Workbench (https://www.humanconnectome.org/software/connectome-workbench), ANTs DenoiseImage, ANTs N4BiasFieldCorrection, ExploreDTI (exploredti.com), TrackVis (trackvis.org), R (4.3.1), Rstudio (Version 2023.06.1+524), the MICE package (Buuren & Groothius-Oudshoorn, 2011), the lavaan package (Rosseel & Y. lavaan, 2012), the lme4 package (Bates et al.2015)

For manuscripts utilizing custom algorithms or software that are central to the research but not yet described in published literature, software must be made available to editors and reviewers. We strongly encourage code deposition in a community repository (e.g. GitHub). See the Nature Portfolio guidelines for submitting code & software for further information.

Data

Policy information about availability of data

All manuscripts must include a data availability statement. This statement should provide the following information, where applicable:

- Accession codes, unique identifiers, or web links for publicly available datasets
- A description of any restrictions on data availability
- For clinical datasets or third party data, please ensure that the statement adheres to our policy

The raw data are available from the corresponding authors on request. The processed data necessary to reproduce the central findings in the manuscript are available at our github page. Custom R scripts were used to analyze and plot all data. Code is available online at our github page.

Research involving human participants, their data, or biological material

Policy information about studies with human participants or human data. See also policy information about sex, gender (identity/presentation), and sexual orientation and race, ethnicity and racism.

Reporting on sex and gender	Gender data were collected based on parent-report to ensure that the sample was balanced. Gender was accounted in all mixed models examining training-related changes.
Reporting on race, ethnicity, or other socially relevant groupings	Ethnicity data were collected to inform on the ethnic composition of the sample and whether it is representative. This included the following categories: Asian, Black, Mixed / multiple ethnic groups; White. Socioeconomic status (SES) was assessed based on employment and education of both parents. SES was used as a covariate in some analyses to test the generalisability of results.
Population characteristics	A total of 262 typically developing children were recruited for the study (6.03-13.31 years; Age M = 8.97, Females = 52.84%) from schools within Greater London in the United Kingdom. Ethnic composition of our sample was as follows: Asian = 14.65%; Black = 3.18%; Mixed/multiple ethnic groups = 17.20%; White = 64.33%; Other = 0.63%.
Recruitment	Recruitment occurred via contacting over 2000 schools in the Greater London area. Out of those schools 20 ended up participating from a diverse range of London boroughs. Information material was disseminated amongst parents of participating schools and only those children whose parents / carers had signed them up ended up taking part.
Ethics oversight	UCL ethics committee

Note that full information on the approval of the study protocol must also be provided in the manuscript.

Field-specific reporting

Please select the one below that is the best fit for your research. If you are not sure, read the appropriate sections before making your selection.

☐ Life sciences ☒ Behavioural & social sciences ☐ Ecological, evolutionary & environmental sciences

For a reference copy of the document with all sections, see nature.com/documents/nr-reporting-summary-flat.pdf

Behavioural & social sciences study design

All studies must disclose on these points even when the disclosure is negative.

Study description	The study used a longitudinal quantitative study design. Participants were randomly assigned into either an experimental group training cognitive control (through inhibition) or to an active control group training response speed. All participants undertook 8-weeks training intervention. Behavioral and neuroimaging data were collected at T0 (pre-training), T1 (immediate post-training), and T2 (1-year post-training) to examine training effects.
Research sample	In order to examine if training cognitive control affects other domains in typically developing children, a total of 262 typically developing children were recruited for the study (6.03-13.31 years; Age M = 8.97, Females = 52.84%) from schools within Greater London in the United Kingdom effectively as a convenience sample. After exclusion of incomplete data, our sample consisted of 235 children (6.03-13.31 years; Age M = 8.97, Females = 51.91%). Ethnic composition of our sample was as follows: Asian = 14.65%; Black = 3.18%; Mixed/multiple ethnic groups = 17.20%; White = 64.33%; Other = 0.63%. Although there was a positive skew in SES (M = 1.64; on a scale of 1-5 where 1 is the highest score attainable), our further analyses showed that the results still hold in lower SES participants. The UCL ethics committee approved the study (Protocol number: 12271/001). In accordance with this, written consent was obtained from parents and assent from children after a description of the study was provided.
Sampling strategy	Convenience sampling was employed in the study. Sampling occurred via contacting over 2000 schools in the Greater London area. Out of those schools 20 ended up participating from a diverse range of London boroughs. Information material was disseminated amongst parents of participating schools and only those children whose parents / carers had signed them up ended up taking part. Power calculations estimated that to obtain even a small group by session interaction effect of f = 0.1 with a power of 0.95 at an alpha Bonferroni corrected for the present number of measures (19; corrected alpha = 0.0025) requires a minimal sample size of 119

	participants. The present sample is almost twice that and therefore amply powered.
Data collection	Data collection was divided into 4 main phases. After an initial baseline data collection phase at pre-test, the 8-week computerized intervention was administered. This was followed up by a post-test and finally, a 1-year-follow-up. Behavioural, questionnaires and neural data (i.e. at pre-test, post-test, 1-year-follow-up) were collected to examine independent near- and far-transfer changes. Due to disruptions to in-person testing during the Covid-19 pandemic, no MRI was obtained at 1-year follow-up. The 3 assessment timepoints took place onsite at the author's laboratory: before the training (T0), after the training (T1), and one-year follow-up (T2). Note that, due to the outbreak of the COVID-19 pandemic in March 2020, some participants completed one or more assessment timepoints online from home. A total of 12 researchers were involved in data collection and in overseeing training taking part at school. Researchers were blind to the training condition of participants. Participants were blind to the training condition they had been assigned to.
Timing	Data collection started in May 2019 and ended in May 2021
Data exclusions	Participants were excluded on the basis of formal diagnoses of neurodevelopmental disorders as well as a safety protocol for neuroimaging (e.g. metal in the body; claustrophobia).
Non-participation	Retention was 71.24% from pre- to post-test and 99.40% from post-test to 1-year follow-up.
Randomization	Children were randomly assigned to an experimental group training cognitive control (through inhibition) or to an active control group training response speed, with groups matched for gender and age, school and class based on mean matching. Matching was performed by an experimenter not involved in testing.

Reporting for specific materials, systems and methods

We require information from authors about some types of materials, experimental systems and methods used in many studies. Here, indicate whether each material, system or method listed is relevant to your study. If you are not sure if a list item applies to your research, read the appropriate section before selecting a response.

Materials & experimental systems

n/a	Involved in the study
☒	☐ Antibodies
☒	☐ Eukaryotic cell lines
☒	☐ Palaeontology and archaeology
☒	☐ Animals and other organisms
☒	☐ Clinical data
☒	☐ Dual use research of concern
☒	☐ Plants

Methods

n/a	Involved in the study
☒	☐ ChIP-seq
☒	☐ Flow cytometry
☐	☒ MRI-based neuroimaging

Magnetic resonance imaging

Experimental design

Design type	Task-related fMRI with event related-design; Resting State fMRI
Design specifications	The SSRT task used outside of the scanner was employed for task-related fMRI where 2 runs (54 trials each, jittered ITI = 2200 to 3000ms) was administered. Each run lasted approximately 5 minutes each, and were acquired using T2*-weighted echo-planar imaging. Each trial started with the presentation of a fixation cross of 1250ms. During the task, participants were asked to press the left arrow key when seeing the 'go' signal (i.e. a honey pot) on the left side of the screen and the down arrow key when the signal appeared on the right side. On 25% of the trials (i.e. a 'stop' trial), a picture of bees was presented after the honey pot. This served as the 'stop' signal. The stop signal delay (SSD) started at 200ms, decreased by 50ms after a successful 'stop' trial, and increased by 50ms after an unsuccessful 'stop' trial. Resting State fMRI consisted of 1 run, which lasted 5 minutes. Participants were instructed to observe a fixation cross presented on a screen, which could be viewed through a mirror attached to the head coil.
Behavioral performance measures	Button press response were recorded during imaging acquisition. Fixed statistical effects were calculated at the individual level by modeling each trial condition ('stop' successful, 'stop' unsuccessful, 'go' successful and 'go' unsuccessful) with a box-car function convolved with the canonical hemodynamic response function.

Acquisition

Imaging type(s)	functional, structural, diffusion MRI
Field strength	3.0T
Sequence & imaging parameters	Task-related fMRI were acquired using T2*-weighted echo-planar imaging (EPI; TR = 1.25s, TE = 35.2 ms, sequential acquisition, 60 slices of 2 x 2 x 2 mm3 voxels, field of view 1696 x 1696, 106 × 106 matrix, in-plane resolution 2 mm). Resting State were completed in a 5-mins run (212 EPI volumes, 60 slices/volume, voxel size 2 x 2 x 2 mm3, TR = 1250

ms, TE = 35.2 ms, flip angle = 65º). High-resolution T1-weighted images were acquired using a magnetisation-prepared rapid gradient-echo sequence (MP-Rage; TR = 2.30s TE = 2.98ms, flip angle = 8°, slices = 1 x 1 x 1 mm3 voxels, field of view 256 x 256). A field map scan was acquired (1 EPI volume, 72 slices/volume, voxel size 2 x 2 x 2 mm3, TR = 8000 ms, TE = 66 ms, flip angle = 90º).

Area of acquisition: Whole brain

Diffusion MRI: ☒ Used ☐ Not used

Parameters: The data is multi shell. 100 diffusion weighted volume directions (50 x b-value of 1,000 s/mm2, 50 x b-value of 2,000 s/mm2) and 5 volumes without diffusion gradient. Cardiac gating was not used.

Preprocessing

Preprocessing software:
Task-related fMRI: SPM12
Resting state fMRI: the ABCD-HCP pipeline (https://github.com/DCAN-Labs/abcd-hcp-pipeline)
Structural MRI: FreeSurfer (Version 6.0.0)
Diffusion MRI: ExploreDTI

Normalization:
Task-related fMRI: Each individual's functional scans were realigned to correct for head motion by initial realignment to first image and second realignment to mean image). The realigned scans were co-registered with anatomical T1-weighted images and spatially normalized to the standard Montreal Neurological Institute (MNI) space by resampling to a voxel size of 2 × 2 × 2 mm3. Normalized images were smoothed with an 8-mm Gaussian filter.
Resting state fMRI: The normalization takes 5 stages: First, the PreFreeSurfer stage normalises the anatomical data, including denoising and bias field correction. Second, the FreeSurfer stage prepares cortical surfaces from the normalised anatomical data, and performs registration to a standard surface template. Third, the PostFreeSurfer stage transforms the volumes to a standard volume template, and the surfaces to a standard surface space. Fourth, the fMRIVolume stage performs processing of the functional data, including bias field correction and registration to the standard volume template. Fifth, the fMRISurface stage maps the normalised functional volumes to the standard surface template.
Structural MRI: sulcal and gyral features across individual subjects were aligned by morphing each subject's brain to an average spherical representation that accurately matches cortical thickness measurements across participants while minimizing metric distortion. A 10mm Gaussian smoothing kernel was applied to data to reduce measurement noise but preserve the capacity for anatomical localizations.
Diffusion MRI: The data was corrected for head motion, eddy current distortions and echo planar imaging distortions and the b-matrix was rotated. Remaining outliers due to head motion and cardiac pulsation were excluded using REKINDLE. The tensor model was fitted to the data using a nonlinear least square fitting procedure. DTI scalar maps, including fractional anisotropy and mean diffusivity were calculated and exported.

Normalization template:
fMRI: standard Montreal Neurological Institute space (MNI152)
Structural MRI: fsaverage5

Noise and artifact removal:
fMRI: To reduce movement-related artifacts, six motion parameters were included as regressors, as well as an additional regressor to model images that were corrupted due to head motion >1.5 mm and were replaced by interpolations of adjacent images (<10% of participant's data).
Diffusion MRI: A whole brain tractography algorithm using Euler integration and the following settings was applied: step size = 0.5 mm, fractional anisotropy threshold ≥0.15, and angle threshold ≤35.

Volume censoring:
MRI: All scans were manually visually inspected for quality, and if necessary, segmentation was manually corrected in FreeSurfer. Four independent inspectors conducted these checks, and one final inspector performed a final inspection of all scans. After corrections, scans were re-segmented using FreeSurfer.
DTI: The connections were dissected in regions corresponding to the putamen and the frontal lobes, providing measures for the fronto-putamen connections. All dissections were completed after ensuring intrarater reliability.

Statistical modeling & inference

Model type and settings: Effects were calculated with a stick function convolved with the canonical hemodynamic response function. Random effects (subject) were used at first level and fixed effects (condition) were used at second level.

Effect(s) tested:
Task-related fMRI: Repeated measures ANOVA was conducted at the group level, with the stop successful condition and go successful condition entered as fixed effects, and a subject factor entered as random effects.
Structural: Cortex-wide linear models were used to assess the effects of training group and time, controlling for age and sex

Specify type of analysis: ☐ Whole brain ☐ ROI-based ☒ Both

Anatomical location(s):
Functional activity from the right inferior frontal gyrus were selected from the probabilistic Harvard-Oxford atlas (thresholded at 20%, center of mass: 51, 28, 8).
Using the Desikan-Killiany atlas, cortical thickness was extracted from the right IFG (compromising of the right pars triangularis, pars opercularis and pars orbitalis) to look at the specific interaction within this region.

Statistic type for inference

(See Eklund et al. 2016)

fMRI: FWE-corrected at cluster-level of $p < .05$, based on an uncorrected height threshold of $p < .001$.
Structural MRI: Findings from the surface-based analyses were controlled for multiple comparisons using random field theory. This reduced the chance of reporting a family-wise error (FWE). The cluster-defining threshold was set to $p<.01$ and the FWE to $p<.05$.

Correction

Family wise error-corrections (FWE) at $p < .05$ were applied to the data.

Models & analysis

n/a	Involved in the study
☐	☒ Functional and/or effective connectivity
☒	☐ Graph analysis
☒	☐ Multivariate modeling or predictive analysis

Functional and/or effective connectivity

fMRI: Time-series of RSFC data was extracted using the Gordon-333 parcellation, which includes 333 parcels (ROIs) that cover the whole cortical surface. Parcels were grouped for the networks of interest (frontoparietal network, FPN; cingulo-opercular network, CON) and correlations across parcels within each network were run. The mean Z-score were calculated across all correlations within each network and an RSFC value (Z-score) were obtained for each network of interest, participant and timepoint.
DTI: Reliability was tested using a two-way mixed intraclass correlation coefficient (ICC).For all tracts, the ICC for single measures reached >0.90. For each tract fractional anisotropy and mean diffusivity were calculated. These measures reflect the structural integrity of the white matter connection and may indicate microstructural differences such as myelination, axonal integrity and how compact fiber bundles are.
